# A Phenomics-Based Strategy Identifies Loci on *APOC1*, *BRAP*, and *PLCG1* Associated with Metabolic Syndrome Phenotype Domains

**DOI:** 10.1371/journal.pgen.1002322

**Published:** 2011-10-13

**Authors:** Christy L. Avery, Qianchuan He, Kari E. North, Jose L. Ambite, Eric Boerwinkle, Myriam Fornage, Lucia A. Hindorff, Charles Kooperberg, James B. Meigs, James S. Pankow, Sarah A. Pendergrass, Bruce M. Psaty, Marylyn D. Ritchie, Jerome I. Rotter, Kent D. Taylor, Lynne R. Wilkens, Gerardo Heiss, Dan Yu Lin

**Affiliations:** 1 Department of Epidemiology, The University of North Carolina at Chapel Hill, Chapel Hill, North Carolina, United States of America; 2 Department of Biostatistics, The University of North Carolina at Chapel Hill, Chapel Hill, North Carolina, United States of America; 3 Carolina Center for Genome Sciences, The University of North Carolina at Chapel Hill, Chapel Hill, North Carolina, United States of America; 4 Information Sciences Institute, University of Southern California, Los Angeles, California, United States of America; 5 Division of Epidemiology, The University of Texas Health Science Center, Houston, Texas, United States of America; 6 Center for Human Genetics, The University of Texas Health Science Center, Houston, Texas, United States of America; 7 Office of Population Genomics, National Human Genome Research Institute, National Institutes of Health, Bethesda, Maryland, United States of America; 8 Fred Hutchinson Cancer Research Center, Seattle, Washington, United States of America; 9 General Medicine Division, Massachusetts General Hospital and Harvard Medical School, Boston, Massachusetts, United States of America; 10 Division of Epidemiology and Community Health, University of Minnesota, Minneapolis, Minnesota, United States of America; 11 Center for Human Genetics Research, Vanderbilt University Medical Center, Nashville, Tennessee, United States of America; 12 Cardiovascular Health Research Unit, Departments of Medicine, Epidemiology, and Health Services, University of Washington, Seattle, Washington, United States of America; 13 Group Health Research Institute, Group Health Cooperative, Seattle, Washington, United States of America; 14 Medical Genetics Institute, Cedars-Sinai Medical Center, Los Angeles, California, United States of America; 15 Cancer Research Center, University of Hawaii, Honolulu, Hawaii, United States of America; Georgia Institute of Technology, United States of America

## Abstract

Despite evidence of the clustering of metabolic syndrome components, current approaches for identifying unifying genetic mechanisms typically evaluate clinical categories that do not provide adequate etiological information. Here, we used data from 19,486 European American and 6,287 African American Candidate Gene Association Resource Consortium participants to identify loci associated with the clustering of metabolic phenotypes. Six phenotype domains (atherogenic dyslipidemia, vascular dysfunction, vascular inflammation, pro-thrombotic state, central obesity, and elevated plasma glucose) encompassing 19 quantitative traits were examined. Principal components analysis was used to reduce the dimension of each domain such that >55% of the trait variance was represented within each domain. We then applied a statistically efficient and computational feasible multivariate approach that related eight principal components from the six domains to 250,000 imputed SNPs using an additive genetic model and including demographic covariates. In European Americans, we identified 606 genome-wide significant SNPs representing 19 loci. Many of these loci were associated with only one trait domain, were consistent with results in African Americans, and overlapped with published findings, for instance central obesity and *FTO*. However, our approach, which is applicable to any set of interval scale traits that is heritable and exhibits evidence of phenotypic clustering, identified three new loci in or near *APOC1*, *BRAP*, and *PLCG1,* which were associated with multiple phenotype domains. These pleiotropic loci may help characterize metabolic dysregulation and identify targets for intervention.

## Author Summary

The metabolic syndrome represents a clustering of metabolic phenotypes (e.g. elevated blood pressure, cholesterol levels, and plasma glucose, as well as abdominal obesity) and is associated with an increased risk of atherosclerosis and type 2 diabetes. Although multiple genes influencing the specific metabolic syndrome components have been reported, few studies have evaluated the genetic underpinnings of the syndrome as a whole. Here, we describe an approach to evaluate multiple clustered traits, which allows us to test whether common genetic variants influence the co-occurrence of one or more metabolic phenotypes. By examining approximately 20,000 European American and 6,200 African American participants from five studies, we show that three regions on chromosomes 12, 19, and 20 are associated with multiple metabolic phenotypes. These genetic variants are highly intriguing candidates that may increase our understanding of the biologic basis of the clustering of metabolic phenotypes and help identify targets for early intervention.

## Introduction

The metabolic syndrome represents metabolic dysregulation expressed as the clustering of several physiologic risk factors and is associated with an increased risk of atherosclerosis and type 2 diabetes [1]. The core metabolic syndrome domains are abdominal obesity, atherogenic dyslipidemia, elevated blood pressure, elevated plasma glucose, a pro-thrombotic state, and a pro-inflammatory state [2], which are represented to varying degrees in commonly used metabolic syndrome scoring systems [3]–[7].

Several lines of evidence support a genetic basis underlying the core metabolic syndrome domains. Measures of metabolic domains cluster in families [8] and heritability estimates range from 16% for systolic blood pressure to 60% for high-density lipoprotein (HDL) cholesterol [9]. Genome-wide association (GWA) studies have also identified common variants in *CETP*, *LPL, APOA5,* and *GCKR* that influence the co-occurrence of metabolic domain phenotypes [10], [11].

Despite evidence of the clustering of metabolic domain phenotypes, current approaches for identifying unifying genetic mechanisms (i.e. pleiotropy) remain largely focused on clinical categories that do not provide adequate etiological information [12]. As an alternative, a phenomics approach that assembles coherent sets of phenotypic features that extend across individual measurements and diagnostic boundaries creates the opportunity for novel genetic investigations of established biological pathways and complements the traditional GWA study or candidate gene-based strategy focused on individual phenotypes [13]–[15]. In addition to making use of existing knowledge on process-related information or pathways, a multi-phenotype phenomics approach also may provide greater statistical power than analyses of individual phenotypes [16] and improve the ability to detect effects of small magnitude [17]. Although several authors have advocated the use of such strategies [15], [18], [19], the approach is implemented infrequently.

This study evaluated evidence of pleiotropy in clustered metabolic domains using data from five well characterized population-based studies composed of approximately 20,000 European American and 6,200 African American participants: the Atherosclerosis Risk in Communities (ARIC) study, the Coronary Artery Risk Development in Young Adults (CARDIA) study, the Cardiovascular Health Study (CHS), the Framingham Heart Study (FHS), and the Multi-Ethnic Study of Atherosclerosis (MESA). Six phenotype domains (atherogenic dyslipidemia, vascular dysfunction, vascular inflammation, pro-thrombotic state, central obesity and elevated plasma glucose) encompassing 19 quantitative traits were examined. After dimension reduction, we applied a statistically efficient and computationally feasible multivariate approach that related the phenotype domains to 250,000 imputed SNPs. Our approach, which is applicable to studies of heritable, clustered interval scale outcomes, identified several genome-wide significant loci associated with multiple phenotype domains, which may help characterize metabolic dysregulation and identify targets for intervention.

## Results

After excluding duplicate samples (N = 56), first- and second-degree relatives (N = 1,152) in all studies except the family-based Framingham Heart Study, and individuals identified as genetic outliers (N = 20), there were 19,468 European American and 6,287 African American Candidate Gene Association Resource Consortium (CARe) participants available for analysis. As expected, CARDIA participants (mean age: 25 years) had better cardiovascular health profiles, including lower low density lipoprotein concentrations, markers of vascular inflammation, and blood pressure levels when compared to the older cohorts (Tables S1, S2, S3, S4, S5).

**Figure 1:**
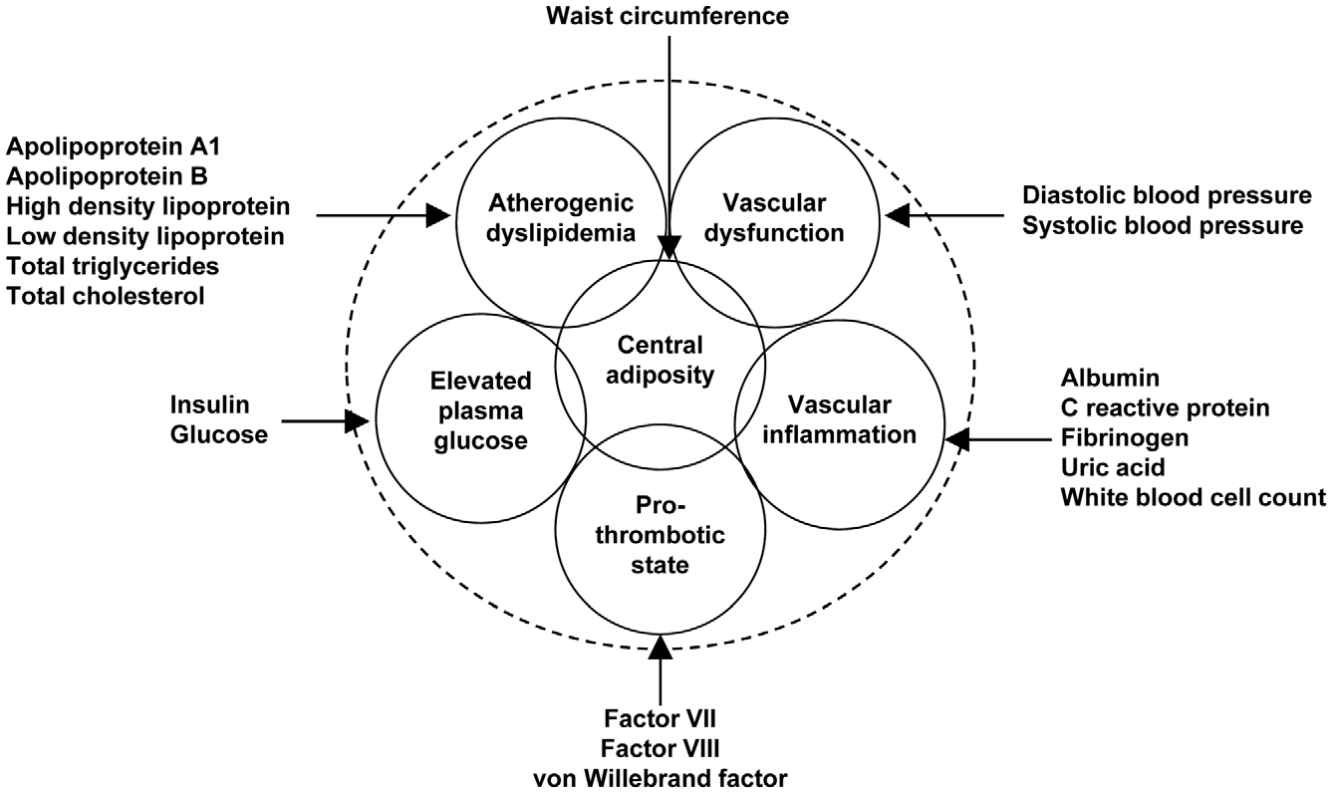
Variables used to characterize six metabolic syndrome domains.

Eight principle components were used to characterize the six metabolic syndrome trait domains (Figure 1): one principal component each for vascular dysfunction, elevated plasma glucose, pro-thrombotic state and central obesity and two principal components for atherogenic dyslipidemia and vascular inflammation. Correlation between the principal components, which served as the eight phenotypes of interest, was modest and consistent across studies and racial groups. As an example, race- specific results from the ARIC Study are presented in Tables S6, S7.

ARIC and CARDIA were the only studies with full phenotype data for all 19 of the variables used to define the metabolic trait domains. Although apolipoprotein A1 and B measurements were unavailable in three cohorts, the high correlations with high-density and low-density lipoprotein concentrations (*r*>0.70 in ARIC data, Tables S8, S9) suggested that all five cohorts provided similar atherogenic dyslipidemia phenotypes. A similarly high correlation was observed between von Willebrand factor and factor VIII in the ARIC data, implying a common pro-thrombotic phenotype in studies missing either measurement. The modest correlation between systemic markers of inflammation in the MESA study, which did not measure white blood cell count and uric acid concentration, suggests that this study may contribute a slightly different vascular inflammation phenotype. The MESA study also did not assay factor VII, suggesting that this study also contributed a somewhat different pro-thrombotic phenotype. However, a sensitivity analysis excluding pro-thrombotic and inflammation principal components estimated in the MESA study yielded comparable results.

**Figure 2:**
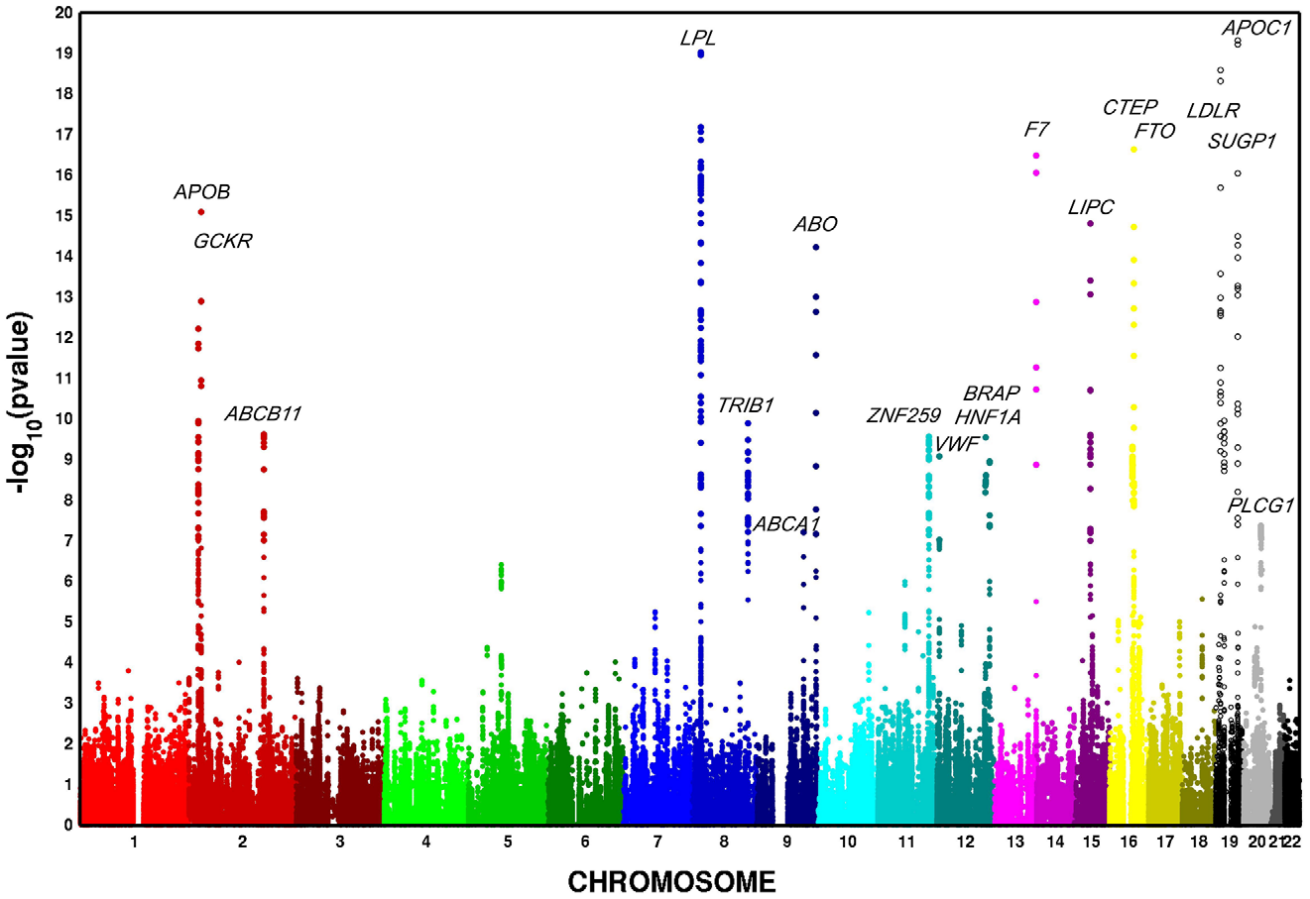
Multivariate association scan of the metabolic syndrome in n = 19,468 European American participants from five cohorts. Y-axis *P*–values are truncated at 1×10^−20^.

**Figure 3:**
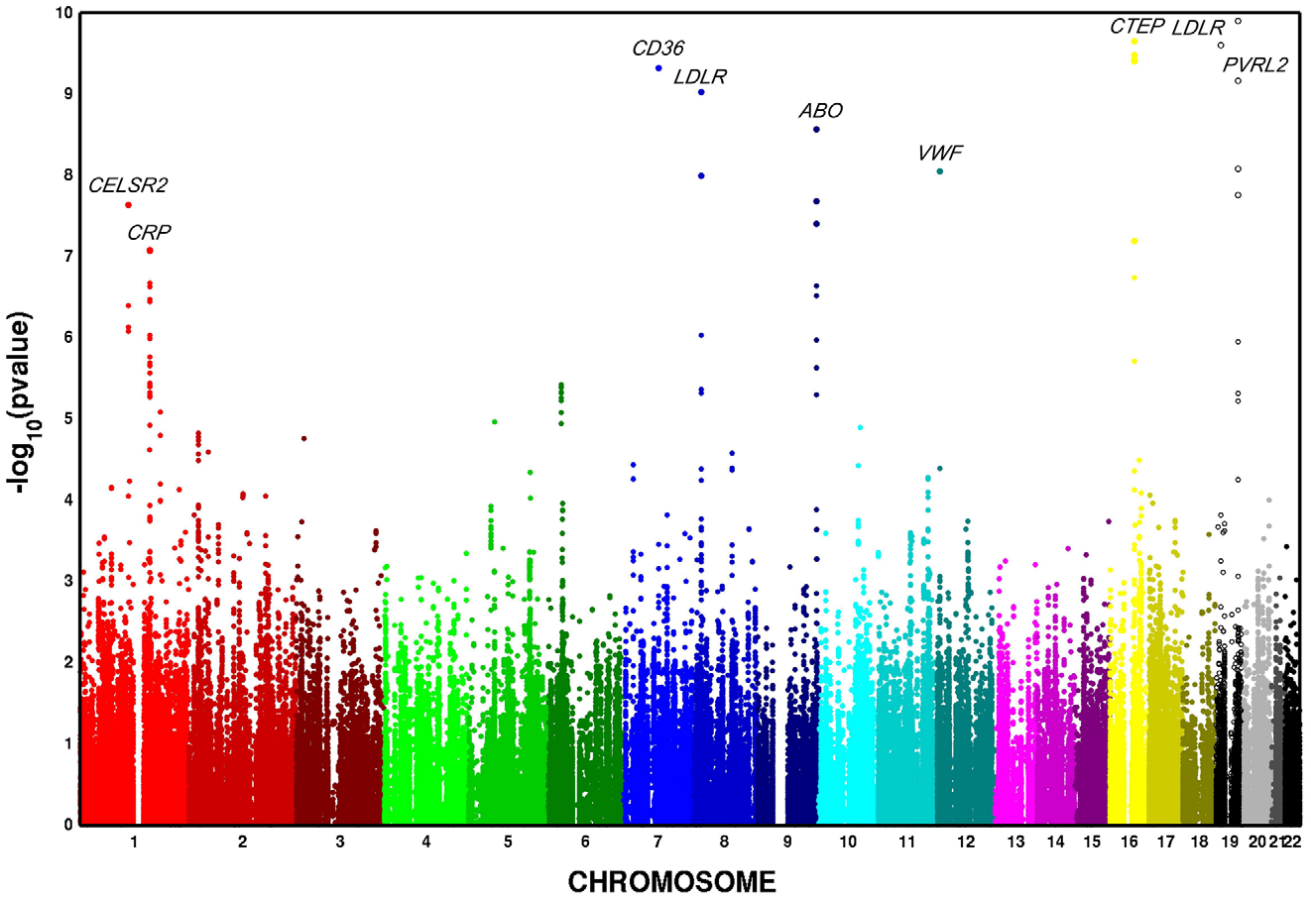
Multivariate GWAS of metabolic syndrome in n = 6,287 African American participants from four cohorts. Y-axis *P*–values are truncated at 1×10^−20^.

In European Americans, we identified 606 SNPs representing 19 loci that were associated with at least one metabolic trait domain (Table 1, Figure 2) at the genome-wide significance level (*P*<2.13×10^−7^; the SNP with the lowest *P* – value chosen if multiple significant SNPs were identified for a given locus) and these results were consistent across the multiple large cohorts (Table S10 and Figure S1). Several of these loci overlapped results in African Americans (Table 2, Figure 3), including associations with *LPL, ABO, VWF, CTEP,* and *LDLR*. In addition to these 19 loci, we also identified 15 additional secondary signals in European Americans, defined as genome-wide significant SNPs (the SNP with the lowest *P* – value chosen if multiple significant SNPs were identified for a given locus) in very low linkage disequilibrium (LD) (r^2^<0.05) with the most significant SNP and within the same 1,000-kb region (Table S11). To verify the independent contributions of these additional loci, we performed a conditional analysis using the most significant SNP at each significant locus as a covariate. Thirteen of these signals remained significant, including one *APOC1* variant, after adjusting for the primary signals.

**Table 1:** Associations for 19 known, confirmed, or possible new loci for metabolic syndrome trait dimensions in n = 19,468 European Americans from five studies.

								Univariate *P*							
SNP^a^	Gene	Distance from gene (kb)	Chr	Position (build 36)	Coded AF	Alleles^b^	MV *P*	Vascular dysfunction	Elevated plasma glucose	AtherogenicDyslipidemia		VascularInflammation		Pro-thrombotic state	Central Obesity
										1	2	1	2		
rs1713222	*APOB*	∼99.5	2	21124828	0.17	A/G	6.1×10^−13^	0.81	0.30	8.7×10^−13^	0.026	0.27	0.65	0.29	0.89
rs1260326	*GCKR*	NSYN	2	27584444	0.43	T/C	8.1×10^−16^	0.14	1.2×10^−8^	2.6×10^−3^	0.95	0.027	0.43	0.016	0.41
rs579060	*ABCB11*	Intronic	2	169491285	0.65	T/G	2.4×10^−10^	0.28	4.1×10^−6^	0.57	6.5×10^−3^	0.013	0.022	0.17	0.054
rs301	*LPL*	Intronic	8	19861214	0.76	T/C	9.5×10^−20^	0.025	8.0×10^−3^	1.4×10^−8^	7.4×10^−18^	0.27	0.15	0.88	0.035
rs2954021	*TRIB1*	100.6	8	126551259	0.50	A/G	1.3×10^−10^	0.051	0.12	1.2×10^−11^	0.035	0.28	0.87	0.094	0.29
rs2575876	*ABCA1*	Intronic	9	106705560	0.26	A/G	6.2×10^−8^	0.21	0.78	0.032	1.2×10^−07^	0.49	0.26	0.26	0.037
rs687621	*ABO*	Intronic	9	135126886	0.66	A/G	<1.0×10^−300^	0.84	0.015	4.9×10^−4^	0.093	0.15	0.81	<1×10^−300^	0.55
rs964184	*ZNF259*	3′UTR	11	116154127	0.86	C/G	5.5×10^−22^	0.89	0.47	1.2×10^−10^	1.8×10^−12^	0.13	0.38	0.61	0.70
rs216318	*VWF*	Intronic	12	6009522	0.09	A/C	1.6×10^−7^	0.61	0.68	0.053	0.36	0.78	0.68	1.9×10^−10^	0.44
rs11065987°	*BRAP*	∼9.9	12	110556807	0.54	A/G	2.9×10^−10^	2.2×10^−4^	0.016	0.72	3.1×10^−3^	0.86	0.17	0.078	9.7×10^−3^
rs7979473	*HNF1A*	Intronic	12	119904643	0.41	A/G	1.1×10^−9^	0.17	0.58	0.16	1.4×10^−3^	2.6×10^−8^	0.55	0.012	0.64
rs510335	*F7*	5′UTR	13	112807756	0.13	T/G	1.0×10^−35^	0.88	0.88	0.84	0.28	0.20	0.57	4.9×10^−49^	0.92
rs397923	*LIPC*	∼35.1	15	56479410	0.42	A/T	1.6×10^−15^	0.02	0.063	0.19	7.1×10^−18^	0.91	0.57	0.73	0.11
rs9923233	*FTO*	Intronic	16	52376699	0.41	C/G	4.9×10^−10^	0.071	4.3×10^−5^	0.75	0.67	0.098	0.63	0.086	1.7×10^−12^
rs247616	*CETP*	∼6.7	16	55547091	0.33	T/C	8.3×10^−72^	0.48	0.53	7.1×10^−6^	5×10^−61^	0.42	0.49	0.46	0.86
rs6511720	*LDLR*	Intronic	19	11063306	0.12	T/G	8.3×10^−28^	0.75	0.76	5.2×10^−30^	0.034	0.93	0.38	0.68	0.46
rs10401969	*SUGP1*	Intronic	19	19268718	0.93	T/C	1.1×10^−10^	0.17	0.16	1.4×10^−8^	0.37	2.2×10^−3^	0.86	0.67	0.78
rs4420638°	*APOC1*	∼0.32	19	50114786	0.82	A/G	1.7×10^−57^	0.87	8.7×10^−4^	1×10^−31^	0.91	5×10^−12^	0.38	0.015	1.2×10^−6^
rs753381°	*PLCG1*	NSYN	20	39230879	0.45	T/C	4.3×10^−8^	0.26	0.03	1.2×10^−3^	0.012	0.16	0.025	0.28	0.01

a The most significant SNP for each locus is presented. ^b^Coded allele is listed first. AF, allele frequency. ^c^Novel locus. Chr, chromosome. MV, multivariate. NSYN, non-synonymous. UTR, untranslated region.

**Table 2:** Associations for 9 known, confirmed, or possible new loci for metabolic syndrome trait dimensions in 6,287 African American participants from four studies.

								Univariate *P*							
SNP	Gene	Distance from gene (kb)	Chr	Position (build 36)	Coded AF	Alleles^a^	MV *P*	Vascular dysfunction	Elevated plasma glucose	AtherogenicDyslipidemia		VascularInflammation		Pro-thrombotic state	Central Obesity
										1	2	1	2		
rs12740374	*CELSR2*	3′UTR	1	109619113	0.25	T/G	3.6×10^−13^	0.26	0.80	8.0×10^−16^	0.30	0.32	0.20	0.058	0.27
rs2592887	*CRP*	∼29.1	1	157919563	0.45	T/C	8.4×10^−8^	0.62	0.64	0.77	0.17	2.8×10^−7^	0.79	0.90	7.0×10^−3^
rs3211938	*CD36*	NSYN	7	80138385	0.91	T/G	4.8×10^−10^	0.39	0.083	0.72	9.4×10^−10^	9.0×10^−4^	0.19	4.8×10^−3^	0.76
rs10096633	*LPL*	∼32.9	8	19875201	0.41	T/C	1.8×10^−12^	0.60	0.59	0.88	1.1×10^−15^	0.66	0.31	0.68	0.99
rs8176693	*ABO*	Intronic	9	135127478	0.10	T/C	6.1×10^−75^	0.20	0.05	0.54	0.12	0.29	0.55	<1×10^−300^	0.098
rs2229446	*VWF*	NSYN	12	5973333	0.18	T/C	9.0×10^−9^	0.29	0.46	0.78	0.30	0.45	0.26	6.3×10^−11^	0.91
rs247616	*CETP*	∼6.7	16	55547091	0.26	T/C	1.9×10^−23^	0.64	0.80	0.20	3.5×10^−23^	0.02	0.34	0.49	0.26
rs6511720	*LDLR*	Intronic	19	11063306	0.14	T/G	2.5×10^−10^	0.94	0.10	9.5×10^−12^	0.096	0.069	0.60	0.27	0.92
rs7254892	*PVRL2*	Intronic	19	50081436	0.07	A/G	1.3×10^−10^	0.22	0.46	2.0×10^−11^	0.093	0.88	0.88	0.09	0.30

**a** Coded allele is listed first. AF, allele frequency. Chr, chromosome. MV, multivariate. NSYN, non-synonymous. UTR, untranslated region.

### Previously identified loci associated with single metabolic trait domains

The strongest signal for both European American and African American participants was located on chromosome 9 in the *ABO* gene (*P*<1.0×10^−300^ and *P* = 6.1×10^−75^, respectively). These signals overlap earlier findings between factor VIII and von Willebrand factor with *ABO* [20]. Nine additional loci in European Americans and eight loci in African Americans demonstrated effects limited to one metabolic syndrome trait domain that have already been reported in the GWA literature and are therefore not considered further: *ABCA1, APOB*, *CD36, CELSR2, CETP, CRP, F7, LDLR, LIPC, PVRL2, TRIB1, VWF,* and *ZNF259.*

### Previously identified loci associated multiple trait domains

Six loci were *associated* with at least two trait domains in European Americans: *GCKR, ABCB11, LPL, HNF1A, FTO,* and *SUGP1,* results which overlap published associations identified through GWA studies for individual trait components. For example, several GWA studies have identified associations between *GCKR* and elevated plasma glucose [21], atherogenic dyslipidemia [22], and vascular inflammation [23]–[25]. *GCKR* is a plausible unifying mechanism for the clustering of metabolic domains, as the protein inhibits glucokinase, the predominant glucose phosphorylating enzyme [26]. *HNF1A*, which encodes the transcription factor hepatocyte nuclear factor (HNF)-1a, also suggests a common pathogenic background, as previous GWA studies have identified associations with atherogenic dyslipidemia [27], vascular inflammation [28], and type 2 diabetes [29]. Of note, *FTO* was the only previously identified and consistently replicated obesity locus we identified.

### Candidate genes at new loci

**Figure 4:**
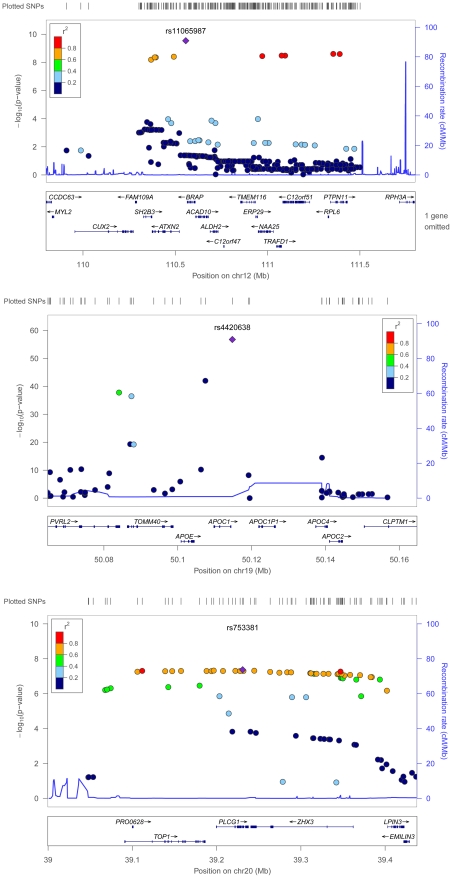
Regional association plots for metabolic syndrome trait dimensions associated with *APOC1*, *BRAP*, and *PLCG1*. Positions are from NCBI build 36 and recombination rates are estimated from HapMap phase II CEU data. SNPs are represented by *circles*, and the *large blue diamond* is the SNP with the lowest *P-*value. Circle color represents correlation with the top SNP: *blue* indicates weak correlation and *red* indicates strong correlation. Recombination rate is plotted in the background and known genes in the region are shown at the bottom of the plot.

The strongest new pleiotropic signal in European Americans was for rs4420638 (*P* 1.7×10^−57^), located approximately 0.32 kilobases (kb) downstream of *APOC1* and associated with elevated plasma glucose (*P* = 8.7×10^−4^), atherogenic dyslipidemia (1×10^−31^), vascular inflammation (*P* = 5×10^−12^), and central obesity (*P* = 1.2×10^−6^). Although associations between *APOC1* with atherogenic dyslipidemia [22], [30], [31] and vascular inflammation [32], [33] have been reported and replicated in the GWA study literature, we consider it a novel locus due to the strong and previously unreported associations with elevated plasma glucose and central obesity. Localizing this signal is challenging, as the region contains a 48-kb gene cluster that also includes the *APOE* and pseudo-*APOC’* genes [34]. However, the modest levels of linkage disequilibrium (Figure 4), the presence of a second signal (Table S11), studies which demonstrate that mice overexpressing human *APOC1* show a marked reduction in the update of fatty acids into adipocytes [35], and the fact the physiological role of *APOC1* is less well established than *APOE, APOB,* and *APOA1* [36] all support further evaluation and fine mapping of *APOC1.*

The second new locus was rs11065987 (*P* = 2.9×10^−10^), located approximately 9.9 kb upstream of *BRAP* and associated with atherogenic dyslipidemia (3.1×10^−3^), vascular dysfunction (2.2×10^−4^), and central obesity (9.7×10^−3^). Initial reports suggested that the *BRAP* protein binds the breast cancer suppressor protein *BRCA1* [37]. *BRAP* is also known to modulate mitogen activated protein kinase signaling [38], an established cell survival, growth, differentiation, transformation, and proinflammatory pathway [39].

The GWA study literature provides few clues that link *BRAP* with metabolic trait domains, as associations have only been identified for alanine aminotransferase [24] and esophageal cancer [40], both in populations of Japanese descent. However, the recombination rate (cM/Mb) is low from approximately 110.3 Mb to 111.5 Mb (Figure 4) and this extended region includes loci associated with type 1 diabetes [41], [42], vascular dysfunction [43], and waist-hip ratio [44]. The *ATXN2* gene, located 27 kb from the index SNP, is an intriguing candidate gene. Expansion of a CAG repeat in the ataxin-2 protein causes the neurodegenerative disease spinocerebellar ataxia type 2. However, instead of a neurodegenerative phenotype, *ATXN2-*deficient rodents exhibited phenotypes characterized by abdominal obesity, insulin resistance, and marked hepatosteatosis (i.e. lipid accumulation in the liver) [45]. Linkage studies of obesity in humans have also associated this region with BMI and total fat percentage [46].

A third genome-wide significant signal was identified for rs753381 (*P* = 4.3×10^−8^), a missense mutation in *PLCG1* that results in a change from an isoleucine to a threonine. *PLCG1* encodes a protein that catalyzes the formation of inositol 1,4,5-trisphosphate and diacylglycerol from phosphatidylinositol 4,5-bisphosphate and plays an important role in the intracellular transduction of receptor-mediated tyrosine kinase activators [47]. Few epidemiologic studies of *PLCG1* or neighboring genes have been published. However, mice nullizygous for *PLCG1* stop growing mid-gestation and show no evidence of vasculogenesis [48]. Vasculogenesis has been associated with insulin resistance [49], plasminogen activator inhibitor-1(PAI-1) concentration [50], hyperglycemia, and adiponectin levels [51]. This suggests that *PLCG1* may contribute to the clustering of metabolic domains in a more subtle manner, such as through small alterations in the structure of the *PLCG1* protein. Thus, the missense mutation we identified would serve as a highly intriguing candidate SNP for further study.

## Discussion

In this study composed of approximately 20,000 European American and 6,200 African American participants, we identified three new loci associated with multiple metabolic trait domains: *APOC1, BRAP,* and *PLCG1*. These loci were in or near genes previously associated with atherogenic dyslipidemia, vascular inflammation, type I diabetes, vascular dysfunction, and central adiposity. No previous genome-wide or gene-centric studies examining evidence for pleiotropy in metabolic domains has detected these loci at genome-wide significant levels.

The pathogenesis of the clustering of metabolic phenotypes remains poorly understood, although it is likely that a sedentary lifestyle, combined with dietary patterns and genetic susceptibility factors, contribute. Candidate genes associated with metabolic syndrome phenotypes largely reflect current knowledge of established pathways regulating obesity, free fatty acid metabolism, insulin sensitivity, lipid metabolism, and inflammation. Although candidate gene and GWA studies have successfully identified loci influencing variation in these pathways, studies examining genetic factors influencing the co-occurrence of metabolic phenotypes are limited. Additionally, those that examine the clustering of syndromic components using the pre-defined clinical cutpoints are largely inconsistent or inconclusive. This general lack of success may reflect ongoing controversy over metabolic syndrome definitions, leading to phenotypic heterogeneity and inconsistent genetic findings across studies [52]. The utility of studying the syndrome as a binary entity as opposed to a series of component traits is also debated [12], especially since the dichotomization of interval scale traits will discard information.

Methods for examining evidence of pleiotropy remain uncommon in the GWA literature and most likely reflect the lack of methodologies and software that are scalable to GWA studies. In this paper, we present a statistically efficient and computational feasible approach to testing for pleiotropy on a genome-wide scale. Our method is applicable to population-based and family studies and identified several associations that would not have been identified through typical univariate analyses. The approach presented herein is also not limited to metabolic phenotypes. Instead, our method could be applied to any set of interval scale traits that are heritable and exhibit evidence of phenotypic clustering.

Although alternative analytic approaches were available, for example estimating principal components for all traits simultaneously, we focused on the phenotype clusters presented in Figure 1. First, evaluating the nineteen phenotypes of interest as six domains of interest is biologically plausible given evidence of phenotypic clustering. It was also easier to interpret principal components that were derived in separate phenotype domains rather than components estimated simultaneously. Additionally, estimating principal components within each phenotype domains ensured that each domain was sufficiently represented in the analysis.

Challenges to the approach presented herein include careful phenotype curation, made more difficult by the inclusion of 19 traits across multiple cohorts that were not measured with a common protocol. Only the ARIC and CARDIA studies had full phenotype information on all 19 traits and CHS was the only study with all traits measured during a single visit. The use of a multivariate phenotype comprised of 19 variables also limited the number of contributing cohorts and the identification of replication cohorts, as few studies have such comprehensive phenotypic data. Nonetheless, we were able to identify approximately 25,000 participants from studies that used standardized, comparable protocols and many of the associations were consistent across cohorts.

Further challenges that are not unique to large scale genetic studies incorporating a phenomics approach include the consistency of results across populations defined by age, race, sex, or other demographic characteristics. For example, the three new loci identified in the European American population were not detected in the African American population. Given a modest sample size of 6,287 participants it is difficult to determine whether an inability to generalize results to the African American population reflects different patterns of LD, varying environmental contexts, or limited statistical power. Variation in mean age between contributing cohorts, which ranged from 25 years in the CARDIA study to 72 years in the CHS, could introduce additional heterogeneity, as associations between metabolic phenotypes have been shown to diminish with age [53]. Finally, marked variation in the prevalence of the metabolic syndrome by gender, regardless of clinical definition, suggest the possibility of sex-specific metabolic syndrome effects [54]. Analyses that examine modification by sex, age, and other important clinical covariates are therefore warranted.

Our use of the IBC array, which is composed of variants implicated in cardiovascular, inflammatory, hemostasis/coagulation, and metabolic pathways, was beneficial in that it allowed us to leverage the wealth of information on pathways implicated in metabolic disturbances while reducing multiple testing penalties. Admittedly this approach was limited in that it potentially excludes novel pathways not captured by the IBC chip. Although imputation allowed us to increase the number of variants, genome-wide approaches might identify additional pleiotropic loci.

In summary, our results support phenomics as a complementary approach that leverages phenotypic variation for the evaluation of pleiotropy, a clear limitation of existing studies examining the metabolic syndrome using clinical definitions. Our approach, which is applicable to studies of heritable, clustered interval scale outcomes, also takes advantage of the wealth of phenotype data available in longitudinal cohort studies as well as emerging analytical and bioinformatics approaches. Ultimately, these results support the presence of genetic variants with pleiotropic effects on adiposity, inflammation, glucose regulation, dyslipidemia, vascular dysfunction and thrombosis. Such loci may help characterize metabolic dysregulation and identify targets for intervention.

## Materials and Methods

### Study population

This study arose from a collaboration between investigators from two National Institute of Health funded consortia examining the genetic basis of common complex diseases: the Population Architecture using Genomics and Epidemiology (PAGE) study, a National Human Genome Research Institute funded effort examining the epidemiologic architecture of common genetic variation that have been reproducibly associated with human diseases and traits [55] and the CARe Consortium [56], a National Heart, Lung, and Blood Institute-supported resource for genetic analyses examining cardiovascular phenotypes. Briefly, PAGE investigators participating in the phenomics working group wanted to extend existing efforts examining evidence for pleiotropy in approximately 300 replicated genetic variants [57] to include a more comprehensive evaluation of common SNPs. A collaboration between PAGE and CARe investigators was therefore initiated, and used data from five CARe studies of European American and African American with adequate phenotype data: ARIC, CARDIA, CHS, FHS, and MESA. All participating institutions and CARe sites obtained Institutional Review Board approval for this study. Additional information on the participating CARe studies is provided in Text S1.

### Genotyping

The Institute for the Translational Medicine and Therapeutics (ITMAT)-Broad-CARe (IBC) genotyping array [58] was used to evaluate approximately 2,100 genes related to cardiovascular, inflammatory, hemostasis/coagulation, and metabolic phenotypes and pathways. The IBC array tagging approach was designed to capture maximal genetic information for both common and lower frequency SNPs (<5% minor allele frequency (MAF)) in HapMap as well as European American and African American populations. The array included 49,320 SNPs, 15,000 of which were gene variants not present in HapMap. Additional details of the SNP selection and tagging approach are given in Text S1.

Imputation of untyped and missing SNP genotypes was performed using MACH 1.0.16. [59] For the European samples, phased haplotypes from the CEU founders of HapMap 2 were used as reference. For African American populations, a combined CEU+YRI reference panel was created that includes SNPs segregating in both CEU and YRI, as well as SNPs segregating in one panel and monomorphic and non-missing in the other. Imputation for the IBC array was performed in two steps. First, individuals with pedigree relatedness or cryptic relatedness were filtered. A subset of individuals was randomly extracted from each panel and used to generate recombination and error rate estimates for the corresponding sample. Second, these rates were used to impute all sample individuals across the entire reference panel. Before cleaning, there were an average of 246,740 (range: 245,816, 247,505) and 227,224 (range: 225,111, 229,061) imputed SNPs in the European American and African American study populations, respectively. Imputation results were then filtered at an imputation quality limit of 0.30 and a MAF threshold of 0.01, yielding 235,077 (95.3% of total) and 227,222 (96.2% of total) SNPs for analysis in European American and African American participants, respectively.

### Phenotypes

The clustered risk factors of interest were characterized as a six-domain phenotype: atherogenic dyslipidemia, vascular dysfunction, vascular inflammation, pro-thrombotic state, elevated plasma glucose, and central obesity (Figure 1). These domains were constructed *a priori* based on a review of literature examining clustering in metabolic phenotypes, placing specific emphasis on the National Cholesterol Education Program’s Adult Treatment Panel III report [4], [60]. Nineteen variables were then selected to represent one of the six domains with preference for variables measured in at least four of the contributing cohort studies or variables that were highly correlated with available measures. Measurement protocols for each variable by study are provided in Table S21. We assessed normality, and transformations were used when variables exhibited excessive skewness or kurtosis as determined by numerical summary information and visual inspection of histograms and normal probability plots. Dimension reduction using principal components analysis was then performed for each phenotype domain separately in each race/ethnic and study population. For example, principal components for the vascular inflammation domain were calculated using the following traits: albumin, C reactive protein, fibrinogen, uric acid, and white blood cell count. Principal components were chosen so that>55% of the variance for each domain was explained (Tables S12, S13, S14, S15, S16, S17, S18, S19, S20). This threshold was chosen because all of the first (waist circumference, pro-thrombotic state, elevated plasma glucose, and vascular dysfunction) and the sum of first and second (vascular inflammation and atherogenic dyslipidemia) principal components exceeded 55% across all studies and racial/ethnic groups.

### Statistical methods

For each phenotype, we fit a linear regression model relating the phenotype to the SNP genotype under the additive mode of inheritance; the model includes environmental variables (i.e., age, sex and study center) as well as the first ten principal components from EIGENSTRAT to adjust for population substructure [61]. Ten population substructure components were included because each component was associated with at least one of the eight phenotypes of interest in at least one study. If the SNP genotype is not associated with any phenotype domain, then the regression coefficients for the SNP genotype are zero in all eight linear models. We tested this global null hypothesis by constructing a multivariate test statistic based on the joint distribution of the score statistics from the eight linear models, which accounted for the correlation between the eight phenotypes. We chose the score statistic because it is computationally efficient and numerically stable. The test statistic is referred to the chi-squared distribution with eight degrees of freedom. The genome-wide significance level was set as *P*<2.13×10^−7^ (i.e. 0.05/235,077). Q-Q plots by race are not presented, as our use of a gene-centric array highly enriched for metabolic loci complicated the identification of markers with low prior probabilities of association (i.e. “null markers”) for all phenotypes of interest. The data from each cohort were analyzed separately and the results were combined via meta-analysis as described in Text S2. All analyses were stratified by race and were performed in SAS 9.1 and C++. Further details are given in the Text S2.

## Supporting Information

Figure S1 Forest plots of univariate effect estimates and 95% confidence intervals for eight metabolic dimensions in n = 19,468 European American participants from five cohorts.
(TIF)

Table S1 Baseline characteristics of ARIC Study participants (N = 11,757) by race.
(DOC)

Table S2 Baseline characteristics of CARDIA Study participants (N = 2,712) by race.
(DOC)

Table S3 Baseline characteristics of CHS Study participants (N = 4,627) by race.
(DOC)

Table S4 Baseline characteristics of Framingham Heart Study participants (N = 2,789).
(DOC)

Table S5 Baseline characteristics of MESA Study participants (N = 3,870) by race.
(DOC)

Table S6 Pearson correlation coefficient estimates for 8 principal components used to characterize the six metabolic domains in n = 9,068 European American ARIC participants.
(DOC)

Table S7 Pearson correlation coefficient estimates for 8 principal components used to characterize the six metabolic domains in n = 2,689 African American ARIC participants.
(DOC)

Table S8 Pearson correlation coefficient estimates for 19 phenotypes used to characterize the six metabolic domains in n = 9,068 European American ARIC participants.
(DOC)

Table S9 Pearson correlation coefficient estimates for 19 phenotypes used to characterize the six metabolic domains in n = 2,689 African American ARIC participants.
(DOC)

Table S10 Overall and study-specific multivariate *P-*values for 19 known, confirmed, or possible new loci for metabolic trait dimensions in n = 19,468 European American from five studies.
(DOC)

Table S11 Fifteen potential secondary signals for metabolic trait dimensions in n = 19,468 European Americans from five studies.
(DOC)

Table S12 Percent variance explained by principal components used to characterize the metabolic trait dimensions, estimated in n = 9,068 European American ARIC participants.
(DOC)

Table S13 Percent variance explained by principal components used to characterize the metabolic trait dimensions, estimated in n = 2,712 African American ARIC participants.
(DOC)

Table S14 Percent variance explained by principal components used to characterize the metabolic trait dimensions, estimated in n = 1,433 CARDIA European American participants.
(DOC)

Table S15 Percent variance explained by principal components used to characterize the metabolic trait dimensions, estimated in n = 1,279 CARDIA African American participants.
(DOC)

Table S16 Percent variance explained by principal components used to characterize the metabolic trait dimensions, estimated in n = 3,892 European American CHS participants.
(DOC)

Table S17 Percent variance explained by principal components used to characterize the metabolic trait dimensions, estimated in n = 735 African American CHS participants.
(DOC)

Table S18 Percent variance explained by principal components used to characterize the metabolic trait dimensions, estimated in n = 2,789 European American FHS participants.
(DOC)

Table S19 Percent variance explained by principal components used to characterize the metabolic trait dimensions, estimated in n = 2,286 European American MESA participants.
(DOC)

Table S20 Percent variance explained by principal components used to characterize the metabolic trait dimensions, estimated in n = 1,584 African American MESA participants.
(DOC)

Table S21 Measurement protocols for 19 phenotypes used to measure metabolic trait dimensions by study.
(DOC)

Text S1 Supplemental methods.
(DOC)

Text S2 Participating studies.
(DOC)
