# Supplementary material for: A Phenomics-Based Strategy Identifies Loci on APOC1, BRAP, and PLCG1 Associated with Metabolic Syndrome Phenotype Domains
Source: PLoS Genet. 2011 Oct 13;7(10):e1002322. doi: 10.1371/journal.pgen.1002322 (PMC3192835; doi:10.1371/journal.pgen.1002322)
Supplement: Table S1 — Baseline characteristics of ARIC Study participants (N = 11,757) by race. (DOC) [file pgen.1002322.s002.doc]

| **TABLE S1. Baseline characteristics of ARIC Study participants (N=11,757) by race.** | | | |
| --- | --- | --- | --- |
| **Characteristic^a^** | | **African Americans**  **(N = 2,689)** | **European**  **American**  **(N = 9,068)** |
| **Age (years)** | | 53 (5.8) | 54 (5.7) |
| **Female (%)** | | 63.2 | 53.6 |
| **Atherogenic dyslipidemia** | |  |  |
|  | Apolipoprotein A1 (mg/dl) | 139.0 (32.1) | 131.1 (30.9) |
|  | Apolipoprotein B (mg/dl) | 92.6 (30.8) | 93.6 (28.1) |
|  | High density lipoprotein (mg/dl) | 54.8 (17.5) | 50.7 (16.9) |
|  | Low density lipoprotein (mg/dl) | 137.9 (42.9) | 137.1 (37.6) |
|  | Total triglycerides (mg/dl) | 114.3 (82.2) | 136.3 (92.5) |
|  | Total cholesterol (mg/dl) | 215.0 (45.1) | 214.4 (40.7) |
| **Vascular dysfunction** | |  |  |
|  | Diastolic blood pressure (mmHg) | 79.4 (12.0) | 71.5 (10.0) |
|  | Systolic blood pressure (mmHg) | 128.1 (20.8) | 118.3 (16.9) |
| **Vascular inflammation** | |  |  |
|  | Albumin (gm/dl) | 3.8 (0.30) | 3.9 (0.25) |
|  | C reactive protein (µG/ml)^b^ | 6.0 (8.4) | 4.1 (6.0) |
|  | Fibrinogen (mg/dl) | 319.4 (70.9) | 296.2 (60.8) |
|  | Uric acid (mg/dl) | 6.3 (1.7) | 5.9 (1.5) |
|  | White blood cell count (x1,000 cubic mm) | 5.6 (1.8) | 6.3 (2.0) |
| **Pro-thrombotic state** | |  |  |
|  | Factor VII (%) | 117.0 (30.6) | 118.8 (29.1) |
|  | Factor VIII (%) | 147.2 (47.2) | 125.2 (34.4) |
|  | Von Willebrand factor (%) | 133.9 (55.8) | 111.8 (42.6) |
| **Elevated Plasma Glucose** | |  |  |
|  | Glucose (mg/dl) | 117.4 (55.4) | 105.0 (30.5) |
|  | Insulin (pmol/L) | 20.6 (43.7) | 12.1 (15.9) |
| **Central Obesity** | |  |  |
|  | Waist circumference (cm) | 99.4 (15.1) | 96.0 (13.3) |
| **ATPIII Metabolic Syndrome Classification** | | | |
| N. components^c^ | |  |  |
|  | 0 | 23.7 | 14.4 |
|  | 1 | 28.4 | 27.1 |
|  | 2 | 21.6 | 27.7 |
|  | 3 | 15.7 | 19.7 |
|  | 4 | 8.2 | 8.4 |
|  | 5 | 2.4 | 2.7 |
| Metabolic syndrome^d^ | | 26.3 | 30.8 |
| ^a^Data are percentages for dichotomous characteristics and means (standard deviation) for continuous variables. ^b^Measured at the fourth study visit. ^c^Components defined as: waist circumference > 102 cm in males or >88 cm in females, triglycerides ≥ 150 mg/dl, High density lipoprotein cholesterol < 40 mg/dl in males or < 50 mg/dl in females, blood pressure ≥ 130/≥85 mm Hg, and fasting glucose ≥ 110 mg/dL. ^d^Defined as having ≥ 3 components. | | | |
