# Supplementary material for: A Phenomics-Based Strategy Identifies Loci on APOC1, BRAP, and PLCG1 Associated with Metabolic Syndrome Phenotype Domains
Source: PLoS Genet. 2011 Oct 13;7(10):e1002322. doi: 10.1371/journal.pgen.1002322 (PMC3192835; doi:10.1371/journal.pgen.1002322)
Supplement: Table S2 — Baseline characteristics of CARDIA Study participants (N = 2,712) by race. (DOC) [file pgen.1002322.s003.doc]

| **TABLE S2. Baseline characteristics of CARDIA Study participants (N=2,712) by race.** | | | |
| --- | --- | --- | --- |
| **Characteristic**^a^ | | **African Americans**  **(N =1,279)** | **European**  **American**  **(N =1,433)** |
| **Age (years)** | | 24 (3.8) | 25 (3.3) |
| **Female (%)** | | 59.3 | 53.2 |
| **Atherogenic dyslipidemia** | |  |  |
|  | Apolipoprotein A1 (mg/dl) | 141.6 (21.5) | 136.3 (19.1) |
|  | Apolipoprotein B (mg/dl) | 90.8 (24.0) | 90.9 (23.4) |
|  | High density lipoprotein (mg/dl) | 54.4 (13.1) | 51.9 (12.5) |
|  | Low density lipoprotein (mg/dl) | 111.2 (31.8) | 108.8 (29.3) |
|  | Total triglycerides (mg/dl) | 66.2 (35.4) | 78.1 (57.0) |
|  | Total cholesterol (mg/dl) | 178.9 (34.1) | 176.3 (31.8) |
| **Vascular dysfunction** | |  |  |
|  | Diastolic blood pressure (mmHg) | 69.1 (9.9) | 68.5 (9.1) |
|  | Systolic blood pressure (mmHg) | 111.4 (10.9) | 109.3 (10.8) |
| **Vascular inflammation** | |  |  |
|  | Albumin (gm/dl) | 4.5 (0.32) | 4.7 (0.30) |
|  | C reactive protein (µG/ml)^b^ | 3.7 (6.8) | 2.1 (4.3) |
|  | Fibrinogen (mg/dl) ^d^ | 226.4 (49.4) | 204.2 (45.8) |
|  | Uric acid (mg/dl) | 5.1 (1.4) | 5.3 (1.4) |
|  | White blood cell count (x1,000 cubic mm) | 5.9 (2.0) | 6.2 (1.7) |
| **Pro-thrombotic state** | |  |  |
|  | Factor VII (%)^c,d^ | 102.3 (44.5) | 101.8 (39.2) |
|  | Factor VIII (%)^c,e^ | 101.7 (39.2) | 90.1 (31.0) |
|  | Von Willebrand factor (%)^d^ | 104.0 (43.6) | 90.7 (33.8) |
| **Elevated Plasma Glucose** | |  |  |
|  | Glucose (mg/dl) | 81.6 (15.7) | 83.1 (13.2) |
|  | Insulin (µU/ml) | 12.3 (8.9) | 9.3 (6.6) |
| **Central Obesity** | |  |  |
|  | Waist circumference (cm) | 78.6 (12.1) | 77.0 (10.4) |
| **ATPIII Metabolic Syndrome Classification** | | | |
| N. components^f^ | |  |  |
|  | 0 | 61.8 | 60.4 |
|  | 1 | 30.0 | 28.7 |
|  | 2 | 6.3 | 8.4 |
|  | 3 | 1.4 | 2.1 |
|  | 4 | 0.43 | 0.4 |
|  | 5 | 0.1 | 0 |
| Metabolic syndrome^g^ | | 1.9 | 2.5 |
| ^a^Data are percentages for dichotomous characteristics and means (standard deviation) for continuous variables. ^b^Measured at year 7. ^c^Measured on cohort sample. ^d^Measured at year 2. ^e^Measured at year 5. ^f^Components defined as: waist circumference > 102 cm in males or >88 cm in females, triglycerides ≥ 150 mg/dl, High density lipoprotein cholesterol < 40 mg/dl in males or < 50 mg/dl in females, blood pressure ≥ 130/≥85 mm Hg, and fasting glucose ≥ 110 mg/dL. ^g^Defined as having ≥ 3 components. CARDIA, Coronary Artery Risk Development in Young Adults. | | | |
