# Supplementary material for: A Phenomics-Based Strategy Identifies Loci on APOC1, BRAP, and PLCG1 Associated with Metabolic Syndrome Phenotype Domains
Source: PLoS Genet. 2011 Oct 13;7(10):e1002322. doi: 10.1371/journal.pgen.1002322 (PMC3192835; doi:10.1371/journal.pgen.1002322)
Supplement: Table S3 — Baseline characteristics of CHS Study participants (N = 4,627) by race. (DOC) [file pgen.1002322.s004.doc]

| **TABLE S3. Baseline characteristics of CHS Study participants (N=4,627) by race.** | | | |
| --- | --- | --- | --- |
| **Characteristic^a^** | | **African Americans**  **(N = 735)** | **European**  **American**  **(N = 3,892)** |
| **Age (years)** | | 73 (5.8) | 73 (5.6) |
| **Female (%)** | | 62.5 | 56.1 |
| **Atherogenic dyslipidemia** | |  |  |
|  | Apolipoprotein A1 (mg/dl)^b^ | --- | --- |
|  | Apolipoprotein B (mg/dl)^b^ | --- | --- |
|  | High density lipoprotein (mg/dl) | 57.8 (15.7) | 53.6 (15.7) |
|  | Low density lipoprotein (mg/dl) | 128.3 (36.2) | 130.0 (35.4) |
|  | Total triglycerides (mg/dl) | 115.6 (64.6) | 144.1 (79.6) |
|  | Total cholesterol (mg/dl) | 208.7 (39.2) | 211.5 (39.0) |
| **Vascular dysfunction** | |  |  |
|  | Diastolic blood pressure (mmHg) | 75.0 (11.3) | 70.0 (11.2) |
|  | Systolic blood pressure (mmHg) | 141.8 (22.7) | 135.3 (21.4) |
| **Vascular inflammation** | |  |  |
|  | Albumin (gm/dl) | 3.9 (0.28) | 4.0 (0.29) |
|  | C reactive protein (mg/dl) | 4.5 (6.1) | 3.4 (6.0) |
|  | Fibrinogen (mg/dl) | 345.0 (74.7) | 319.5 (64.4) |
|  | Uric acid (mg/dl) | 5.8 (1.6) | 5.7 (1.5) |
|  | White blood cell count (x1,000 cubic mm) | 5.8 (1.7) | 6.4 (2.0) |
| **Pro-thrombotic state** | |  |  |
|  | Factor VII (%) | 111.2 (27.2) | 125.4 (29.9) |
|  | Factor VIII (%) | 139.3 (43.8) | 120.8 (37.0) |
|  | Von Willebrand factor(%)^b^ | --- | --- |
| **Elevated Plasma Glucose** | |  |  |
|  | Glucose (mg/dl) | 119.3 (53.3) | 109.7 (33.1) |
|  | Insulin (IU/ml) | 20.6 (43.6) | 16.6 (23.0) |
| **Central Obesity** | |  |  |
|  | Waist circumference (cm) | 98.8 (14.2) | 93.8 (12.8) |
| **ATPIII Metabolic Syndrome Classification** | | | |
| N. components^c^ | |  |  |
|  | 0 | 14.5 | 8.7 |
|  | 1 | 26.9 | 26.1 |
|  | 2 | 25.3 | 31.7 |
|  | 3 | 18.7 | 21.7 |
|  | 4 | 10.5 | 8.6 |
|  | 5 | 4.1 | 3.2 |
| Metabolic syndrome^d^ | | 33.3 | 33.5 |
| **^a^**Data are percentages for dichotomous characteristics and means (standard deviation) for continuous variables. ^b^Unavailable. ^c^Components defined as: waist circumference > 102 cm in males or >88 cm in females, triglycerides ≥ 150 mg/dl, High density lipoprotein cholesterol < 40 mg/dl in males or < 50 mg/dl in females, blood pressure ≥ 130/≥85 mm Hg, and fasting glucose ≥ 110 mg/dL. ^d^Defined as having ≥ 3 components. CHS, Cardiovascular Health Study. | | | |
