# Supplementary material for: A Phenomics-Based Strategy Identifies Loci on APOC1, BRAP, and PLCG1 Associated with Metabolic Syndrome Phenotype Domains
Source: PLoS Genet. 2011 Oct 13;7(10):e1002322. doi: 10.1371/journal.pgen.1002322 (PMC3192835; doi:10.1371/journal.pgen.1002322)
Supplement: Table S4 — Baseline characteristics of Framingham Heart Study participants (N = 2,789). (DOC) [file pgen.1002322.s005.doc]

| **TABLE S4. Baseline characteristics of Framingham Heart Study participants (N=2,789).** | | |
| --- | --- | --- |
| **Characteristic^a^** | | **European**  **American**  **(N =2,789)** |
| **Age (years)** | | 35 (9.8) |
| **Female (%)** | | 54.2 |
| **Atherogenic dyslipidemia** | |  |
|  | Apolipoprotein A1 (mg/dl) ^b^ | 144.4 (29.8) |
|  | Apolipoprotein B (mg/dl) ^b^ | 98.1 (26.2) |
|  | High density lipoprotein (mg/dl) ^b^ | 50.3 (15.2) |
|  | Low density lipoprotein (mg/dl) ^b^ | 132.7 (36.3) |
|  | Total triglycerides (mg/dl) ^b^ | 145.6 (107.9) |
|  | Total cholesterol (mg/dl)^b^ | 204.4 (36.2) |
| **Vascular dysfunction** | |  |
|  | Diastolic blood pressure (mmHg) ^b^ | 72.9 (10.6) |
|  | Systolic blood pressure (mmHg)^b^ | 123.2 (19.2) |
| **Vascular inflammation** | |  |
|  | Albumin (gm/dl)^c^ | 4.7 (0.3) |
|  | C reactive protein (mg/L)^d^ | 2.1 (4.3) |
|  | Fibrinogen (mg/dl)^b^ | 304.8 (57.6) |
|  | Uric acid (mg/dl) ^c^ | 5.6(1.4) |
|  | White blood cell count (x1,000 cubic mm) ^c^ | 6.1 (1.6) |
| **Pro-thrombotic state** | |  |
|  | Factor VII (%)^b^ | 100.1 (16.6) |
|  | Factor VIII (%)^e^ | --- |
|  | Von Willebrand factor (%)^b^ | 125.8 (45.1) |
| **Elevated Plasma Glucose** | |  |
|  | Glucose (mg/dl) ^b^ | 99.8 (26.5) |
|  | Insulin (pmol/L)^f^ | 13.5 (10.0) |
| **Central Obesity** | |  |
|  | Waist circumference (cm)^b^ | 92.4 (14.1) |
| **ATPIII Metabolic Syndrome Classification** | | |
| N. components^g^ | |  |
|  | 0 | 26.4 |
|  | 1 | 25.8 |
|  | 2 | 20.8 |
|  | 3 | 16.1 |
|  | 4 | 7.9 |
|  | 5 | 3.1 |
| Metabolic syndrome^h^ | | 27.0 |
| **^a^**Data are percentages for dichotomous characteristics and means (standard deviation) for continuous variables. ^b^Measured at the fifth study visit. ^c^Measured at the first study visit. ^d^Measured at the second study visit. ^e^Unavailable. ^f^Measured at the seventh study visit. ^g^Components defined as: waist circumference > 102 cm in males or >88 cm in females, triglycerides ≥ 150 mg/dl, High density lipoprotein cholesterol < 40 mg/dl in males or < 50 mg/dl in females, blood pressure ≥ 130/≥85 mm Hg, and fasting glucose ≥ 110 mg/dL. ^h^Defined as having ≥ 3 components. | | |
