# Supplementary material for: A Phenomics-Based Strategy Identifies Loci on APOC1, BRAP, and PLCG1 Associated with Metabolic Syndrome Phenotype Domains
Source: PLoS Genet. 2011 Oct 13;7(10):e1002322. doi: 10.1371/journal.pgen.1002322 (PMC3192835; doi:10.1371/journal.pgen.1002322)
Supplement: Table S5 — Baseline characteristics of MESA Study participants (N = 3,870) by race. (DOC) [file pgen.1002322.s006.doc]

| **TABLE S5. Baseline characteristics of MESA Study participants (N=3,870) by race.** | | | |
| --- | --- | --- | --- |
| **Characteristic**^a^ | | **African Americans**  **(N =1,584)** | **European**  **American**  **(N =2,286)** |
| **Age (years)** | | 62 (10.1) | 63 (10.2) |
| **Female (%)** | | 53.9 | 52.2 |
| **Atherogenic dyslipidemia** | |  |  |
|  | Apolipoprotein A1 (mg/dl)^b^ | --- | --- |
|  | Apolipoprotein B (mg/dl) ^b^ | --- | --- |
|  | High density lipoprotein (mg/dl) | 52.4 (15.3) | 52.2 (15.7) |
|  | Low density lipoprotein (mg/dl) | 116.1 (33.3) | 117.0 (30.4) |
|  | Total triglycerides (mg/dl) | 106.2 (59.1) | 137.7 (81.2) |
|  | Total cholesterol (mg/dl) | 189.1 (36.4) | 195.8 (35.6) |
| **Vascular dysfunction** | |  |  |
|  | Diastolic blood pressure (mmHg) | 74.6 (10.2) | 70.1 (10.0) |
|  | Systolic blood pressure (mmHg) | 131.7 (21.6) | 123.6 (20.7) |
| **Vascular inflammation** | |  |  |
|  | Albumin (gm/dl) | 3.3 (16.8) | 1.4 (7.0) |
|  | C reactive protein (mg/L) | 4.9 (7.3) | 3.5 (5.3) |
|  | Fibrinogen (mg/dl) | 361.4 (79.9) | 336.1 (70.4) |
|  | Uric acid (mg/dl) ^b^ | --- | --- |
|  | White blood cell count (x1,000 cubic mm) ^b^ | --- | --- |
| **Pro-thrombotic state** | |  |  |
|  | Factor VII (%)^b^ | --- | --- |
|  | Factor VIII (%) | 179.2 (75.9) | 157.2 (64.6) |
|  | Von Willebrand factor (%) | 156.0 (64.2) | 134.8 (54.5) |
| **Elevated Plasma Glucose** | |  |  |
|  | Glucose (mg/dl) | 100.6 (32.9) | 91.6 (22.3) |
|  | Insulin (pmol/L) | 7.4 (6.5) | 6.0 (4.6) |
| **Central Obesity** | |  |  |
|  | Waist circumference (cm) | 101.3 (14.7) | 98.0 (14.5) |
| **ATPIII Metabolic Syndrome Classification** | | | |
| N. components^c^ | |  |  |
|  | 0 | 21.4 | 12.6 |
|  | 1 | 25.9 | 27.9 |
|  | 2 | 25.4 | 31.9 |
|  | 3 | 18.2 | 18.2 |
|  | 4 | 7.9 | 7.9 |
|  | 5 | 1.2 | 1.5 |
| Metabolic syndrome^d^ | | 27.3 | 27.6 |
| ^a^Data are percentages for dichotomous characteristics and means (standard deviation) for continuous variables. ^b^Unavailabale. ^c^Components defined as: waist circumference > 102 cm in males or >88 cm in females, triglycerides ≥ 150 mg/dl, High density lipoprotein cholesterol < 40 mg/dl in males or < 50 mg/dl in females, blood pressure ≥ 130/≥85 mm Hg, and fasting glucose ≥ 110 mg/dL. ^d^Defined as having ≥ 3 components. MESA, Multi Ethnic Study of Atherosclerosis | | | |
