# Supplementary material for: A Phenomics-Based Strategy Identifies Loci on APOC1, BRAP, and PLCG1 Associated with Metabolic Syndrome Phenotype Domains
Source: PLoS Genet. 2011 Oct 13;7(10):e1002322. doi: 10.1371/journal.pgen.1002322 (PMC3192835; doi:10.1371/journal.pgen.1002322)
Supplement: Table S6 — Pearson correlation coefficient estimates for 8 principal components used to characterize the six metabolic domains in n = 9,068 European American ARIC participants. (DOC) [file pgen.1002322.s007.doc]

| **TABLE S6. Pearson correlation coefficient estimates for 8 principal components used to characterize the six metabolic domains in n=9,068 European American ARIC participants.** | | | | | | | | |
| --- | --- | --- | --- | --- | --- | --- | --- | --- |
|  | Atherogenic  Dyslipidemia | Central adiposity | Vascular dysfunction | Vascular inflammation | | Pro-thrombotic state | Elevated plasma glucose | |
|  | PC2 |  |  | PC1 | PC2 |  |  |  |
| Atherogenic dyslipidemia PC1 | 0 | 0.30 | 0.15 | 0.18 | 0.30 | 0.12 | | 0.29 |
| Atherogenic dyslipidemia PC2 |  | 0.30 | 0.03 | 0.17 | 0.20 | -0.006 | | 0.27 |
| Central adiposity |  |  | 0.28 | 0.33 | 0.29 | 0.18 | | 0.52 |
| Vascular dysfunction |  |  |  | 0.09 | 0.22 | 0.09 | | 0.28 |
| Vascular inflammation PC1 |  |  |  |  | 0 | 0.23 | | 0.22 |
| Vascular inflammation PC2 |  |  |  |  |  | 0.002 | | 0.29 |
| Pro-thrombotic state |  |  |  |  |  |  | | 0.27 |
| ARIC, Atherosclerosis Risk in Communities Study. PC, principal component. | | | | | | | | |
