# Supplementary material for: A Phenomics-Based Strategy Identifies Loci on APOC1, BRAP, and PLCG1 Associated with Metabolic Syndrome Phenotype Domains
Source: PLoS Genet. 2011 Oct 13;7(10):e1002322. doi: 10.1371/journal.pgen.1002322 (PMC3192835; doi:10.1371/journal.pgen.1002322)
Supplement: Table S8 — Pearson correlation coefficient estimates for 19 phenotypes used to characterize the six metabolic domains in n = 9,068 European American ARIC participants. (DOC) [file pgen.1002322.s009.doc]

| **TABLE S8. Pearson correlation coefficient estimates for 19 phenotypes used to characterize the six metabolic domains in n=9,068 European American ARIC participants.** | | | | | | | | | | | | | | | | | | |
| --- | --- | --- | --- | --- | --- | --- | --- | --- | --- | --- | --- | --- | --- | --- | --- | --- | --- | --- |
|  | Atherogenic dyslipidemia | | | | | Central  Adiposity | Vascular dysfunction | | Vascular Inflammation | | | | | Pro-thrombotic state | | | Elevated plasma glucose | |
|  | Apo B | HDL | LDL | TG^a^ | Total Chol | Waist | DBP | SBP | Albumin | CRP | Fibrinogen^a^ | Uric acid | WBC^a^ | F7^a^ | F8^a^ | VWF^a^ | Glucose^b^ | Insulin^a^ |
| Apo A | -0.15 | 0.79 | -0.13 | -0.27 | 0.11 | -0.28 | -0.04 | -0.03 | -0.01 | -0.01 | -0.14 | -0.26 | -0.17 | 0.14 | -0.006 | -0.04 | -0.15 | -0.26 |
| Apo B |  | -0.27 | 0.74 | 0.42 | 0.69 | 0.18 | 0.09 | 0.12 | 0.07 | 0.05 | 0.12 | 0.18 | 0.13 | 0.20 | 0.08 | 0.05 | 0.14 | 0.17 |
| HDL |  |  | -0.21 | -0.51 | 0.06 | -0.40 | -0.09 | -0.08 | -0.001 | -0.04 | -0.16 | -0.38 | -0.23 | 0.07 | -0.05 | -0.08 | -0.23 | -0.38 |
| LDL |  |  |  | 0.26 | 0.92 | 0.13 | 0.07 | 0.09 | 0.13 | 0.02 | 0.14 | 0.11 | 0.05 | 0.16 | 0.05 | 0.05 | 0.05 | 0.09 |
| TG^a^ |  |  |  |  | 0.35 | 0.36 | 0.16 | 0.21 | 0.04 | 0.16 | 0.09 | 0.36 | 0.24 | 0.32 | 0.12 | 0.09 | 0.30 | 0.43 |
| Total Chol |  |  |  |  |  | 0.07 | 0.08 | 0.11 | 0.14 | 0.06 | 0.08 | 0.07 | 0.03 | 0.29 | 0.07 | 0.04 | 0.07 | 0.07 |
| Waist |  |  |  |  |  |  | 0.24 | 0.27 | -0.08 | 0.25 | 0.20 | 0.44 | 0.16 | 0.15 | 0.16 | 0.14 | 0.31 | 0.54 |
| DBP |  |  |  |  |  |  |  | 0.66 | 0.13 | 0.03 | -0.004 | 0.20 | -0.01 | 0.07 | 0.02 | -0.02 | 0.09 | 0.19 |
| SBP |  |  |  |  |  |  |  |  | 0.06 | 0.10 | 0.08 | 0.19 | 0.07 | 0.12 | 0.10 | -0.02 | 0.20 | 0.24 |
| Albumin |  |  |  |  |  |  |  |  |  | -0.20 | -0.18 | 0.09 | -0.11 | -0.04 | -0.15 | -0.12 | -0.005 | 0.02 |
| CRP |  |  |  |  |  |  |  |  |  |  | 0.28 | 0.06 | 0.23 | 0.16 | 0.10 | 0.08 | 0.07 | 0.17 |
| Fibrinogen^a^ |  |  |  |  |  |  |  |  |  |  |  | 0.08 | 0.32 | 0.12 | 0.25 | 0.23 | 0.10 | 0.16 |
| Uric acid |  |  |  |  |  |  |  |  |  |  |  |  | 0.15 | 0.08 | 0.09 | 0.11 | 0.15 | 0.36 |
| WBC^a^ |  |  |  |  |  |  |  |  |  |  |  |  |  | 0.05 | 0.06 | 0.09 | 0.13 | 0.19 |
| F7^a^ |  |  |  |  |  |  |  |  |  |  |  |  |  |  | 0.15 | 0.05 | 0.07 | 0.17 |
| F8^a^ |  |  |  |  |  |  |  |  |  |  |  |  |  |  |  | 0.72 | 0.22 | 0.23 |
| VWF^a^ |  |  |  |  |  |  |  |  |  |  |  |  |  |  |  |  | 0.17 | 0.19 |
| Glucose^b^ |  |  |  |  |  |  |  |  |  |  |  |  |  |  |  |  |  | 0.43 |
| ^a^Natural log transformation applied. ^b^Negative reciprocal transformation applied ARIC, Atherosclerosis Risk in Communities Study. Apo A, apolipoprotein A1. Apo B, Apolipoprotein B. CRP, C reactive protein. DBP, diastolic blood pressure. F7, Factor VII. F8, Factor VIII. HDL, high density lipoprotein. LDL, low density lipoprotein. SBP, systolic blood pressure. TG, triglycerides. Total chol, total cholesterol. VWF, von Willebrand factor. WBC, white blood cell count. | | | | | | | | | | | | | | | | | | |
