# Supplementary material for: A Phenomics-Based Strategy Identifies Loci on APOC1, BRAP, and PLCG1 Associated with Metabolic Syndrome Phenotype Domains
Source: PLoS Genet. 2011 Oct 13;7(10):e1002322. doi: 10.1371/journal.pgen.1002322 (PMC3192835; doi:10.1371/journal.pgen.1002322)
Supplement: Table S10 — Overall and study-specific multivariate P-values for 19 known, confirmed, or possible new loci for metabolic trait dimensions in n = 19,468 European American from five studies. (DOC) [file pgen.1002322.s011.doc]

| **TABLE S10. Overall and study-specific multivariate *P-*values for 19 known, confirmed, or possible new loci for metabolic trait dimensions in n= 19,468 European American from five studies.** | | | | | | | | | | | | | |
| --- | --- | --- | --- | --- | --- | --- | --- | --- | --- | --- | --- | --- | --- |
| **SNP** | **Gene** | **Distance from gene (kb)** | **Chr** | **Position (build 36)** | **Coded**  **AF** | **Alleles^a^** | **Overall**  **MV *P*** |  | **Study-specific MV *P*** | | | | |
|  |  |  |  |  |  |  |  | **ARIC** | | **CARDIA** | **CHS** | **FHS** | **MESA** |
| rs1713222 | *APOB* | ~99.5 | 2 | 21124828 | 0.17 | A/G | 6.1 x 10^-13^ | 5.5 x10^-5^ | | 0.12 | 6.9 x10^-5^ | 0.19 | 0.082 |
| rs1260326 | *GCKR* | NSYN | 2 | 27584444 | 0.43 | T/C | 8.1 x 10^-16^ | 1.3 x10^-4^ | | 0.071 | 2.8 x 10^-11^ | 0.029 | 5.5 x 10^-7^ |
| rs579060 | *ABCB11* | Intronic | 2 | 169491285 | 0.65 | T/G | 2.4 x 10^-10^ | 0.012 | | 4.9 x10^-4^ | 6.5 x 10^-3^ | 3.3 x10^-4^ | 0.066 |
| rs301 | *LPL* | Intronic | 8 | 19861214 | 0.76 | T/C | 9.5 x 10^-20^ | 1.4 x10^-6^ | | 0.11 | 3.7 x 10^-5^ | 6.8 x10^-4^ | 8.9 x 10^-8^ |
| rs2954021 | *TRIB1* | 100.6 | 8 | 126551259 | 0.50 | A/G | 1.3 x 10^-10^ | 2.7 x10^-3^ | | 0.016 | 1.9 x10^-3^ | 0.14 | 0.16 |
| rs2575876 | *ABCA1* | Intronic | 9 | 106705560 | 0.26 | A/G | 6.2 x 10^-8^ | 1.1 x10^-4^ | | 0.37 | 0.62 | 0.25 | 0.032 |
| rs687621 | *ABO* | Intronic | 9 | 135126886 | 0.66 | A/G | < 1.0 x 10^-300^ | < 1.0 x 10^-100^ | | 2.0 x 10^-20^ | 9.7 x 10^-48^ | 8.4 x 10^-13^ | 7.8 x 10^-10^ |
| rs964184 | *ZNF259* | 3'UTR | 11 | 116154127 | 0.86 | C/G | 5.5 x 10^-22^ | 1.1 x10^-5^ | | 4.5 x10^-3^ | 4.3 x 10^-9^ | 9.0 x10^-6^ | 5.8 x10^-4^ |
| rs216318 | *VWF* | Intronic | 12 | 6009522 | 0.09 | A/C | 1.6 x 10^-7^ | 8.9 x10^-5^ | | 0.26 | 0.27 | 0.018 | 0.54 |
| rs11065987 | *BRAP* | ~9.9 | 12 | 110556807 | 0.54 | A/G | 2.9 x 10^-10^ | 1.3 x 10^-6^ | | 0.52 | 3.0 x 10^-3^ | 0.063 | 0.35 |
| rs7979473 | *HNF1A* | Intronic | 12 | 119904643 | 0.41 | A/G | 1.1 x 10^-9^ | 4.7 x10^-4^ | | 0.11 | 2.7 x10^-4^ | 8.9 x 10^-4^ | 0.095 |
| rs510335 | *F7* | 5'UTR | 13 | 112807756 | 0.13 | T/G | 1.0 x 10^-35^ | 4.7 x 10^-7^ | | 0.35 | 1.5 x 10^-47^ | 1.1 x 10^-11^ | 0.54 |
| rs397923 | *LIPC* | ~35.1 | 15 | 56479410 | 0.42 | A/T | 1.6 x 10^-15^ | 6.6 x 10^-8^ | | 0.047 | 0.049 | 0.067 | 2.7 x10^-3^ |
| rs9923233 | *FTO* | Intronic | 16 | 52376699 | 0.41 | C/G | 4.9 x 10^-10^ | 8.0 x 10^-8^ | | 0.92 | 0.023 | 0.064 | 0.80 |
| rs247616 | *CETP* | ~6.7 | 16 | 55547091 | 0.33 | T/C | 8.3 x 10^-72^ | 4.0 x 10^-42^ | | 0.030 | 1.2 x 10^-16^ | 8.5 x 10^-8^ | 6.6 x 10^-7^ |
| rs6511720 | *LDLR* | Intronic | 19 | 11063306 | 0.12 | T/G | 8.3 x 10^-28^ | 4.1 x 10^-10^ | | 7.3 x 10^-3^ | 6.8 x 10^-8^ | 4.5 x 10^-5^ | 0.029 |
| rs10401969 | *SUGP1* | Intronic | 19 | 19268718 | 0.93 | T/C | 1.1 x 10^-10^ | 4.1 X 10^-8^ | | 0.21 | 0.056 | 0.47 | 0.055 |
| rs4420638 | *APOC1* | ~0.32 | 19 | 50114786 | 0.82 | A/G | 1.7 x 10^-57^ | 5.2 X 10^-28^ | | 1.0 x 10^-4^ | 3.9 x 10^-13^ | 3.2 x 10^-6^ | 2.2 x 10^-3^ |
| rs753381 | *PLCG1* | NSYN | 20 | 39230879 | 0.45 | T/C | 4.3 x 10^-8^ | 3.13 x 10^-6^ | | 0.72 | 0.21 | 0.27 | 0.86 |
| ^a^Coded allele is listed first. AF, allele frequency. ARIC, Atherosclerosis Risk in Communities Study. CARDIA, Coronary Artery Risk Development in Young Adults. Chr, chromosome. CHS, Cardiovascular Health Study. FHS, Framingham Heart Study. MESA, Multi-Ethnic Study of Atherosclerosis. MV, multivariate. NSYN, non-synonymous. UTR, untranslated region. | | | | | | | | | | | | | |
