# Supplementary material for: A Phenomics-Based Strategy Identifies Loci on APOC1, BRAP, and PLCG1 Associated with Metabolic Syndrome Phenotype Domains
Source: PLoS Genet. 2011 Oct 13;7(10):e1002322. doi: 10.1371/journal.pgen.1002322 (PMC3192835; doi:10.1371/journal.pgen.1002322)
Supplement: Table S11 — Fifteen potential secondary signals for metabolic trait dimensions in n = 19,468 European Americans from five studies. (DOC) [file pgen.1002322.s012.doc]

| **TABLE S11. Fifteen potential secondary signals for metabolic trait dimensions in n= 19,468 European Americans from five studies.** | | | | | | | | | | | | | | | |
| --- | --- | --- | --- | --- | --- | --- | --- | --- | --- | --- | --- | --- | --- | --- | --- |
| **SNP^a^** | **Gene** | **Distance from gene (kb)** | **Chr** | **Position (build 36)** | **Coded**  **AF** | **Alleles^b^** | **MV *P*** | **Conditional analysis univariate *P*** | | | | | | | |
|  |  |  |  |  |  |  |  | **Vascular dysfunction** | **Elevated plasma glucose** | **Atherogenic**  **Dyslipidemia** | | **Vascular**  **Inflammation** | | **Pro-thrombotic state** | **Central Obesity** |
|  |  |  |  |  |  |  |  |  |  | **1** | **2** | **1** | **2** |  |  |
| rs13414987 | *APOB* | ~71.7 | 2 | 21005313 | 0.24 | A/C | 2.4 x 10^-10^ | 0.87 | 0.20 | 1.1 x 10^-13^ | 0.037 | 0.16 | 0.81 | 0.68 | 0.83 |
| rs3289 | *LPL* | 3'UTR | 8 | 19867472 | 0.97 | T/C | 8.7 x 10^-18^ | 0.15 | 4.2 x 10^-3^ | 4.0 x 10^-8^ | 7 x 10^-18^ | 0.46 | 0.24 | 0.87 | 0.01 |
| rs505922 | *ABO* | Intron | 9 | 135139050 | 0.66 | T/C | 0.74 | 0.34 | 0.25 | 0.59 | 0.76 | 0.095 | 0.82 | 0.35 | 0.71 |
| rs590485 | *SURF6* | ~18.9 | 9 | 135168763 | 0.79 | T/C | < 1 x 10^-300^ | 0.63 | 1.9 x 10^-3^ | 8.2 x 10^-5^ | 0.032 | 0.12 | 0.57 | < 1 x 10^-300^ | 0.55 |
| rs621907 | *MED22* | 3'UTR | 9 | 135197865 | 0.07 | T/C | < 1 x 10^-300^ | 0.53 | 4.7 x 10^-3^ | 8.0 x 10^-5^ | 0.091 | 0.23 | 0.92 | < 1 x 10^-300^ | 0.61 |
| rs3124747 | *C9ORF96* | NSYN | 9 | 135257905 | 0.35 | A/G | < 1 x 10^-300^ | 0.68 | 4.5 x 10^-3^ | 2.9 x 10^-4^ | 0.093 | 0.087 | 0.84 | < 1 x 10^-300^ | 0.48 |
| rs1063856 | *VWF* | NSYN | 12 | 6023795 | 0.64 | T/C | 1.8 x 10^-4^ | 0.95 | 0.78 | 0.13 | 0.36 | 0.69 | 0.89 | 5.7 x 10^-8^ | 0.36 |
| rs555212 | *F7* | ~3.9 | 13 | 112804541 | 0.23 | A/G | 1.8 x 10^-29^ | 0.52 | 0.94 | 0.64 | 0.31 | 0.16 | 0.71 | 1.3 x 10^-35^ | 0.9 |
| rs1077835 | *LIPC* | ~0.50 | 15 | 56510718 | 0.78 | A/G | 1.1 x 10^-14^ | 0.016 | 0.013 | 0.11 | 5.6 x 10^-18^ | 0.94 | 0.44 | 0.47 | 0.049 |
| rs2895432 | *NUP93* | Intron | 16 | 55414456 | 0.09 | A/G | 1.6 x 10^-66^ | 0.18 | 0.30 | 1.2 x 10^-6^ | 1.2 x 10^-66^ | 0.36 | 0.32 | 0.41 | 0.94 |
| rs289714 | *CETP* | Intron | 16 | 55564952 | 0.83 | A/G | 1.3 x 10^-57^ | 0.19 | 0.35 | 7.2 x 10^-6^ | 3.1 x 10^-58^ | 0.20 | 0.39 | 0.49 | 0.91 |
| rs289741 | *CETP* | Intron | 16 | 55574975 | 0.70 | A/G | 1.6 x 10^-66^ | 0.27 | 0.51 | 1.0 x 10^-6^ | 1.1 x 10^-64^ | 0.25 | 0.43 | 0.37 | 0.97 |
| rs4803750 | *BCL3* | ~4.8 | 19 | 49939467 | 0.93 | A/G | 4.2 x 10^-55^ | 0.99 | 3.2 x 10^-5^ | 1.3 x 10^-33^ | 0.90 | 2.4 x 10^-14^ | 0.28 | 9.1 x 10^-3^ | 7.8 x 10^-9^ |
| rs4803760 | *BCAM* | ~9.2 | 19 | 50025674 | 0.23 | T/C | 4.1 x 10^-48^ | 0.88 | 9.4 x 10^-5^ | 9.9 x 10^-32^ | 0.78 | 4.8 x 10^-13^ | 0.53 | 0.01 | 2.9 x 10^-8^ |
| rs445925 | *APOC1* | ~2.2 | 19 | 50107480 | 0.11 | A/G | 5.1 x 10^-54^ | 0.89 | 4.3 x 10^-5^ | 1.3 x 10^-35^ | 0.96 | 3.8 x 10^-14^ | 0.33 | 0.014 | 3.8 x 10^-9^ |
| ^a^The most significant SNP for each locus is presented. ^b^Coded allele is listed first. AF, allele frequency. Chr, chromosome. MV, multivariate. NSYN, non-synonymous. UTR, untranslated region. | | | | | | | | | | | | | | | |
