# Supplementary material for: A Phenomics-Based Strategy Identifies Loci on APOC1, BRAP, and PLCG1 Associated with Metabolic Syndrome Phenotype Domains
Source: PLoS Genet. 2011 Oct 13;7(10):e1002322. doi: 10.1371/journal.pgen.1002322 (PMC3192835; doi:10.1371/journal.pgen.1002322)
Supplement: Table S13 — Percent variance explained by principal components used to characterize the metabolic trait dimensions, estimated in n = 2,712 African American ARIC participants. (DOC) [file pgen.1002322.s014.doc]

| **TABLE S13. Percent variance explained by principal components used to characterize the metabolic trait dimensions, estimated in n=2,712 African American ARIC participants.** | | | | | | |
| --- | --- | --- | --- | --- | --- | --- |
|  | **Percent variance explained** | | | | | |
| **Dimension** | **PC 1** | **PC 2** | **PC 3** | **PC 4** | **PC 5** | **PC 6** |
| Central obesity | 1.0 | --- | --- | --- | --- | --- |
| Elevated plasma glucose | 0.73 | 0.27 | --- | --- | --- | --- |
| Vascular dysfunction | 0.75 | 0.25 | --- | --- | --- | --- |
| Pro-thrombotic state | 0.60 | 0.32 | 0.08 |  | --- | --- |
| Vascular inflammation | 0.32 | 0.24 | 0.18 | 0.15 | 0.11 | --- |
| Atherogenic dyslipidemia | 0.48 | 0.32 | 0.13 | 0.05 | 0.03 | 0.0004 |
| ARIC, Atherosclerosis Risk in Communities Study. PC, principal component. | | | | | | |
