# Supplementary material for: A Phenomics-Based Strategy Identifies Loci on APOC1, BRAP, and PLCG1 Associated with Metabolic Syndrome Phenotype Domains
Source: PLoS Genet. 2011 Oct 13;7(10):e1002322. doi: 10.1371/journal.pgen.1002322 (PMC3192835; doi:10.1371/journal.pgen.1002322)
Supplement: Table S16 — Percent variance explained by principal components used to characterize the metabolic trait dimensions, estimated in n = 3,892 European American CHS participants. (DOC) [file pgen.1002322.s017.doc]

| **TABLE S16. Percent variance explained by principal components used to characterize the metabolic trait dimensions, estimated in n=3,892 European American CHS participants.** | | | | | | |
| --- | --- | --- | --- | --- | --- | --- |
|  | **Percent variance explained** | | | | | |
| **Dimension** | **PC 1** | **PC 2** | **PC 3** | **PC 4** | **PC 5** | **PC 6** |
| Central obesity | 1.0 | --- | --- | --- | --- | --- |
| Elevated plasma glucose | 0.73 | 0.27 | --- | --- | --- | --- |
| Vascular dysfunction | 0.71 | 0.29 | --- | --- | --- | --- |
| Pro-thrombotic state | 0.56 | 0.44 | --- | --- | --- | --- |
| Vascular inflammation | 0.36 | 0.22 | 0.17 | 0.15 | 0.10 | --- |
| Atherogenic dyslipidemia | 0.55 | 0.31 | 0.14 | 0.001 | --- | --- |
| CHS, Cardiovascular Health Study. PC, principal component. | | | | | | |
