# Supplementary material for: A Phenomics-Based Strategy Identifies Loci on APOC1, BRAP, and PLCG1 Associated with Metabolic Syndrome Phenotype Domains
Source: PLoS Genet. 2011 Oct 13;7(10):e1002322. doi: 10.1371/journal.pgen.1002322 (PMC3192835; doi:10.1371/journal.pgen.1002322)
Supplement: Table S19 — Percent variance explained by principal components used to characterize the metabolic trait dimensions, estimated in n = 2,286 European American MESA participants. (DOC) [file pgen.1002322.s020.doc]

| **TABLE S19. Percent variance explained by principal components used to characterize the metabolic trait dimensions, estimated in n=2,286 European American MESA participants.** | | | | | | |
| --- | --- | --- | --- | --- | --- | --- |
|  | **Percent variance explained** | | | | | |
| **Dimension** | **PC 1** | **PC 2** | **PC 3** | **PC 4** | **PC 5** | **PC 6** |
| Central obesity | 1.0 | --- | --- | --- | --- | --- |
| Elevated plasma glucose | 0.71 | 0.29 | --- | --- | --- | --- |
| Vascular dysfunction | 0.76 | 0.24 | --- | --- | --- | --- |
| Pro-thrombotic state | 0.82 | 0.18 | --- | --- | --- | --- |
| Vascular inflammation | 0.49 | 0.33 | 0.18 | --- | --- | --- |
| Atherogenic dyslipidemia | 0.57 | 0.29 | 0.14 | 0.002 | --- | --- |
| MESA, Multi Ethnic Study of Atherosclerosis. PC, principal component. | | | | | | |
