# Supplementary material for: A Phenomics-Based Strategy Identifies Loci on APOC1, BRAP, and PLCG1 Associated with Metabolic Syndrome Phenotype Domains
Source: PLoS Genet. 2011 Oct 13;7(10):e1002322. doi: 10.1371/journal.pgen.1002322 (PMC3192835; doi:10.1371/journal.pgen.1002322)
Supplement: Table S21 — Measurement protocols for 19 phenotypes used to measure metabolic trait dimensions by study. (DOC) [file pgen.1002322.s022.doc]

| **TABLE S21. Measurement protocols for 19 phenotypes used to measure metabolic trait dimensions by study.** | | | | | | |
| --- | --- | --- | --- | --- | --- | --- |
| **Characteristic** | | **ARIC** | **CARDIA** | **CHS** | **FHS** | **MESA** |
| **Blood Collection** | | Participants were asked to fast for 12 hours before their morning clinic appointments, and blood was drawn from an antecubital vein with minimal trauma. The plasma was separated by centrifugation at 4°C and divided into tubes containing EDTA.  Aliquots were stored locally at -70°C until shipping on dry ice to a central lipid laboratory, where they were stored again at -70°C until analysis. Most of the samples were analyzed within 6 weeks of receipt. | Participants were instructed to fast for at least 12 hours before a morning examination. Venous blood was collected using minimal stasis, aliquoted, stored at —70°C, and shipped to central study laboratories for assay. | Subjects came to the clinic after an overnight fast, and blood was obtained on their arrival at the clinic. Samples for lipoprotein lipids were drawn in tubes containing EDTA (final concentration, 1 mg/ml). Plasma was inisolated by centrifugation at 3,000 rpm for 30 minutes at 4°C. Samples were frozen at -70°C and  shipped weekly, on dry ice, to the Central Blood  Laboratory. | After an overnight 12- to 14-hour fast, blood was drawn from the  subjects into tubes containing EDTA (final concentration, 1 mg/mL). | Blood was drawn between 8 AM and 12 noon from participants who had fasted overnight. Then 5 mL blood was placed into each of two Vacutainer tubes containing 3.8% sodium citrate, mixed by repeated inversion, and spun at 3000g for 20 minutes at 4°C in a refrigerated centrifuge. This centrifugation was performed within 10 minutes of blood collection, and within 1 hour the plasma was placed in a -70°C freezer. |
| **Atherogenic dyslipidemia** | |  |  |  |  |  |
|  | **Apolipoprotein A1** | Measured by radioimmunoassay [14,15] | Measured by radioimmunoassay [16]. | N/a | Measured in plasma using a commercially available enzyme-linked imunosorbent assay (Macra Lp(a); strategic Diagnostics Newark, NJ) [17] | N/a |
|  | **Apolipoprotein B** | Measured by radioimmunoassay [14,15] Although processing methods adequately preserve levels, freezing lowers levels by ~7% [18] |  | N/a | Neasured by a noncompetitive enzymelinked  immunosorbent assay (ELISA) using affinitypurified polyclonal anti-apoB antibodies. | N/a |
|  | **High density lipoprotein** | Measured by the method of Warnick [19] | Determined by the dextran sulfate method of Warnick [19] | Measured by an enzymatic method after precipitation of  apolipoprotein B-containing lipoproteins with dextran sulfate/ magnesium sulfate. | Determined  enzymatically on an Abbott Diagnostics ABA-2000 bichromatic  analyzer [20] | Measured by enzymatic methods after dextran sulfate-magnesium precipitation [19] |
|  | **Low density lipoprotein** | Calculated with the formula of Friedewald [21] | Calculated with the formula of Friedewald.[21] | Calculated with the formula of Friedewald [21] | Calculated with the formula of Friedewald [21] | Calculated with the formula of Friedewald [21] |
|  | **Total triglycerides** | Measured by enzymatic methods [22,23] with the use of reagents supplied by Boehringer- Mannheim Biochemical, and were adapted for analysis in the Cobas-Bioanalyzer (Roche). | Determined  enzymatically[24] | Measured by enzymatic methods on an Olympus Demand  System (Olympus Corp., Lake Success, N.Y.). | Determined  enzymatically on an Abbott Diagnostics ABA-2000 bichromatic  analyzer [20] | Determined using  enzymatic procedures on the ABA 200 bichromatic instrument [24] |
|  | **Total cholesterol** |  |  |  |  |  |
| **Vascular dysfunction** | |  |  |  |  |  |
|  | **Diastolic blood pressure** | Three successive measurements were taken after a 5-minute rest with the use of a random-zero sphygmomanometer. The mean of the last 2 measurements was used in this analysis. | Measured after a 5-minute rest using a Hawksley  random-zero sphygmomanometer; three measurements  were recorded 1 minute apart, and the mean of  the last two measurements was analyzed. | Blood pressure  was measured in the right arm with a random-zero  sphygmomanometer with the participant in the sitting position and was defined as the mean of two measurements. | Blood pressures were measured to the nearest 2 mm Hg with a mercury column sphygmomanometer on the left arm after the subject had been seated quietly for 5 minutes. Two readings were averaged to calculate SBP and DBP. | Resting seated blood pressure was measured 3 times using an automated oscillometric sphygmomanometer (Dinamap PRO 100; Critikon, Tampa Bay, Florida); the last 2 measurements were averaged for analysis. |
|  | **Systolic blood pressure** |  |  |  |  |  |
| **Vascular inflammation** | |  |  |  |  |  |
|  | **Albumin** | Coulter DACOS instrument (Coulter Diagnostics, Hialeah, FL, USA) with a bromcresol green colourimetric assay [25] | Measured with a SMACII  continuous-flow analyzer (Technicon Instruments Corp., Tarrytown, NY) [26] | Kodak Ektachem 700 analyzer (Eastman Kodak, Rochester, NY, USA) | Measured by a thin film adaptation of a bromcresol green colorimetric procedure using the  Vitros analyzer [27] | Measured by nephelometry |
|  | **C reactive protein** | Immunoturbidimetric assay using the Siemens (Dade Behring) BNII analyzer (Dade Behring, Deerfield, Il, USA) | Enzyme-linked immunosorbent  assay method with a nephelometrybased  high-throughput assay [28]  . | Measured with an  enzyme linked immunosorbent assay developed at the CHS  central blood laboratory.[29] | Measured with an enzyme immunoassay (Hemagen Diagnostics, Inc). | Measured using the BNII nephelometer (N High Sensitivity CRP; Dade Behring Inc, Deerfield, IL) |
|  | **Fibrinogen** | Thrombin time titration method [30] | Determined by an immunonephelometric  method (Dade Behring Marburg GmbH, Marburg, Germany) on a Behring Nephelometer II analyzer. | Thrombin time titration method [30] | Thrombin time titration method [30] | Determined by an immunonephelometric  method (Dade Behring Marburg GmbH, Marburg, Germany) on a Behring Nephelometer II analyzer. |
|  | **Uric acid** | Uricase method | Uricase method [31] | Measured on the Kodak Ektachem 700 Analyzer | Measured using an autoanalyzer with a  phosphotungstic acid reagent [32] | N/a |
|  | **White blood cell count** | Determined by automated particle counters within 24 hours after venipuncture in local hospital hematology laboratories | Automatic blood cell counters used were: Birmingham, Coulter S+; Chicago, Technicon H-6000; Minneapolis, Coulter S+4; and Oakland, Technicon H-6000 first, then Coulter S+ in 01/1986. | Measured on automated instruments at local hematology laboratories near each field center. | Quantified by  the Coulter counter, | N/a |
| **Pro-thrombotic state** | |  |  |  |  |  |
|  | **Factor VII** | Coagulant activities measured by a one-stage assay using factor VII– and factor VIII–deficient plasma (George King Biomedical, Overland Park, KS). | Coagulant activities were assayed by a one-stage system with reagents from Pacific Hemostasis and  George King Biomedical, Inc. | Measured on the Coag-A-Mate X2 (Organon-Teknika, Durham, NC) using factor VII– and factor VIII–deficient plasma | Antigen levels measured with a commercially available ELISA kit (Diagnostica Stago). | Coagulant activities were assayed by a one-stage system with reagents from Pacific Hemostasis and  George King Biomedical, Inc. |
|  | **Factor VIII** |  |  |  | N/a |  |
|  | **Von Willebrand factor** | Enzyme-linked immunosorbent assay (ELISA) | measured by an enzyme-linked immunosorbent assay obtained from American Bioproducts  Co. [33] | N/a | Measured  using an ELISA technique [34] | Measured by an enzyme-linked immunosorbent assay obtained from American Bioproducts  Co. [33]. |
| **Elevated Plasma Glucose** | |  |  |  |  |  |
|  | **Glucose** | Hexokinase/glucose-6-phosphate dehydrogenase method. | Determined  by the hexokinase-ultraviolet method. | Measured in serum  with the Kodak Ektachem E-700 (Kodak, Rochester,  N.Y.). | Measured with a hexokinase reagent kit (A-Gent Glucose Test, Abbott). | Serum glucose level was measured using a Vitros analyzer (Johnson & Johnson Clinical Diagnostics, Rochester, New York). |
|  | **Insulin** | Radioimmunoassay Insulin Kit; Cambridge Medical Diagnosis, Billerica, MA) | Measured using an immunoassay  method (Diabetes Research Center, University of  Pittsburgh). | Measured with a competitive  radioimmunoassay from Diagnostics Products (Los Angeles, Calif.). | Measured in plasma as total immunoreactive insulin and standardized to serum levels for reporting purposes | Measured by using the Linco Human Insulin Specific Radioimmunoassay kit (Linco Research, Inc., St. Charles, Missouri). |
| **Central Obesity** | |  |  |  |  |  |
|  | **Waist circumference** | Measured at the level of the umbilicus with anthropometric tape to the nearest centimeter with the subjects standing. | Measured laterally midway between the iliac crest and the lowest lateral portion of the rib cage and anteriorly midway between the xiphoid process of the sternum and the umbilicus | Measured using the smallest circumference between the lower ribs and iliac crests | Measured at the level of the umbilicus while the participant was standing. | Measured using a Gulick II anthropometric tape (Sammons Preston, Chicago, Illinois) applied horizontally at the level of the umbilicus and was rounded to the nearest centimeter. |
|  | ARIC, Atherosclerosis Risk in Communities study; CARDIA, Coronary Artery Risk Development in Young Adults; CHS, Cardiovascular Health Study; FHS, Framingham Heart Study; MESA, Multi-Ethnic Study of Atherosclerosis | | | | | |
