# Supplementary material for: A Phenomics-Based Strategy Identifies Loci on APOC1, BRAP, and PLCG1 Associated with Metabolic Syndrome Phenotype Domains
Source: PLoS Genet. 2011 Oct 13;7(10):e1002322. doi: 10.1371/journal.pgen.1002322 (PMC3192835; doi:10.1371/journal.pgen.1002322)
Supplement: Text S1 — Supplemental methods. (DOC) [file pgen.1002322.s023.doc]

**Participating CARe studies**

Atherosclerosis Risk in Communities Study (ARIC): The ARIC study is an ongoing population-based cohort of 15,792 Caucasian and African-American males and females aged 45-64 years at baseline selected using probability sampling from four United States communities (Forsyth County NC, Jackson MS, suburban Minneapolis MN, and Washington County MD) [1]. Participants were recruited in 1987-1989 to examine cardiovascular and pulmonary disease, patterns of medical care, and disease variation over time. Standardized physical examinations and interviewer-administered questionnaires were conducted at baseline, and at three triennial follow-up examinations.

Coronary Artery Risk Development in Young Adults (CARDIA): CARDIA is a longitudinal study of the evolution of coronary heart disease risk, started in 1985–86 in 5,115 African American and Caucasian males and females aged 18–30 years [2]. The CARDIA sample was recruited during 1985–86 primarily from geographically based populations in Birmingham AL, Chicago IL, and Minneapolis MN and, in Oakland, CA, from the membership of the Kaiser-Permanente Health Plan. Examinations after baseline were year 2 (1987–88, n=4624, 90% retention, year 5 (1990–91, n=4352, 85% retention), year 7 (1992–93, n=4086, 80% retention), year 10 (1995–96, n=3950, 79% retention), year 15 (2000–2001, n=3672, 74% retention) and year 20 (2005–06, n=3549, 72% retention).

Cardiovascular Health Study (CHS): CHS is a prospective, longitudinal cohort study of risk factors for cardiovascular disease in the elderly [3]. Starting in 1989, n=5,888 Caucasian and African-American adults aged 65 years or older were sampled from four United States communities (Forsyth County, North Carolina; Sacramento County, California; Washington County, Maryland; and Pittsburgh, Pennsylvania). Participants underwent extensive yearly annual clinical examinations measuring traditional risk factors such as blood pressure and lipids as well as measures of subclinical disease.

Framingham Heart Study (FHS) The FHS started in 1948 with 5,209 randomly ascertained participants from Framingham, MA, who underwent biannual examinations to investigate cardiovascular disease and related risk factors [4,5]. In 1971, the offspring cohort (comprised of 5,124 children of the original cohort and the children’s spouses) and in 2002, the third generation (consisting of 4,095 children of the offspring cohort), were recruited. FHS participants in this study are of European ancestry. This study was limited to members of the offspring cohort.

Multi-Ethnic Study of Atherosclerosis (MESA): MESA was initiated in 2000 to investigate subclinical cardiovascular disease and the risk factors that predict progression to clinically overt cardiovascular disease [6]. The population-based cohort included 6,814 asymptomatic males and females aged 45–84 at study baseline from six field centers (Winston-Salem, NC; St. Paul, MN; Chicago, IL; Los Angeles, CA; New York, NY; Baltimore, MD). MESA investigators enrolled individuals of Caucasian (38%), African American (28%), Hispanic (22%) and (12%) of Chinese descent, although only African American and Caucasian participants were included in the present study.

**Loci and SNP selection for the IBC array**

Several parallel strategies were used to select IBC chip loci [7]. First, a Pubmed search was conducted that evaluated approximately 2,400 published association studies examining cardiovascular phenotypes. Studies were prioritized based on sample size, data quality, and strength of association. Genes implicated in sleep, lung, and blood phenotypes were also nominated and input was solicited directly from established investigators within and outside the IBC consortium. Additionally, pathway-based tools, including Kyoto Encyclopedia of Genes and Genomes (KEGG) [8] and Protein Analysis THrough Evolutionary Relationships (PANTER, http://www.pantherdb.org) and BioCarta (http://www.biocarta.com) were used to identify biologically plausible genes from pathways including lipid metabolism, thrombogenesis, circulation and gas exchange, insulin resistance, metabolism, inflammation, oxidative stress, and apoptosis. Finally, key findings from the animal and population-based literature were provided through early access to several unpublished mouse atherosclerosis expression quantitative trait loci datasets and hypertension, type 2 diabetes, and coronary artery disease GWAS [9,10,11,12].

The IBC array was developed using SNP and linkage disequilibrium information from HapMap as well as resequencing data from SeattleSNPs and the National Institute of Environmental Health Sciences (NIEHS) SNPs [7]. A cosmopolitan tagging approach in which all samples from HapMap were used as a single reference panel was used to produce the optimal union of SNPs to tag the loci of interest at predefined density criteria: minor allele frequency (MAF) as low as 2% and r^2^ = 0.80 for first priority loci (n=435) and as low as MAF of 5% and r^2^ = 0.5 for second priority loci (n=1,349) in HapMAP populations and SeattleSNPs where available. In addition, all non-synonymous SNPs and functional variants with MAF > 1% were included for n=232 loci which were lower interest *a priori* to investigators.

**Genotyping imputation and quality control**

The CEU (NW European descent from Utah) and YRI (Yoruba in Ibadan Nigeria) panels used were from HapMap2 (r22 b36) with monomorphic variants excluded. In total, 2,543,857 and 2,852,179 SNPs were phased in the CEU and YRI panels, respectively. A baseline rsq>=0.4 threshold was applied to the imputation, which was 0.1 more stringent than usual due to an enrichment of poorly imputed SNPs due to the small number of SNPs on the chip

Several quality control (QC) procedures were performed on the genotype data, separately for each cohort. Samples were excluded for sex mismatches, duplicate discordances, or call rates <95%. For each set of duplicates or monozygotic twins, data from the sample with the highest genotyping call rate was retained. SNPs were removed if the call rate was < 95% or HWE *P*<10^–5^ in Caucasians or when monomorphic. (There was no HWE filter for African Americans.) These filters resulted in available data on 47,539 SNPs.

**Principal components calculation**

Principal components were calculated using EIGENSTRAT [13] applied to cleaned CARe IBC genotype data. HapMap populations (CEU, YRI, CHB+JPT) were used as reference (i.e. seed) populations. The top 10 principal components were used as covariates in the regression analyses.

**REFERENCES**

1. ARIC Investigators (1989) The Atherosclerosis Risk in Communities (ARIC) Study: Design and Objectives. The ARIC Investigators. Am J Epidemiol 129: 687-702.

2. Friedman GD, Cutter GR, Donahue RP, Hughes GH, Hulley SB, et al. (1988) CARDIA: study design, recruitment, and some characteristics of the examined subjects. J Clin Epidemiol 41: 1105-1116.

3. Fried LP, Borhani NO, Enright P, Furberg CD, Gardin JM, et al. (1991) The Cardiovascular Health Study: design and rationale. Ann Epidemiol 1: 263-276.

4. Kannel WB, Feinleib M, McNamara PM, Garrison RJ, Castelli WP (1979) An investigation of coronary heart disease in families. The Framingham offspring study. Am J Epidemiol 110: 281-290.

5. Splansky GL, Corey D, Yang Q, Atwood LD, Cupples LA, et al. (2007) The Third Generation Cohort of the National Heart, Lung, and Blood Institute's Framingham Heart Study: design, recruitment, and initial examination. Am J Epidemiol 165: 1328-1335.

6. Bild DE, Bluemke DA, Burke GL, Detrano R, Diez Roux AV, et al. (2002) Multi-ethnic study of atherosclerosis: objectives and design. Am J Epidemiol 156: 871-881.

7. Keating BJ, Tischfield S, Murray SS, Bhangale T, Price TS, et al. (2008) Concept, design and implementation of a cardiovascular gene-centric 50 k SNP array for large-scale genomic association studies. PLoS One 3: e3583.

8. Kanehisa M, Goto S (2000) KEGG: kyoto encyclopedia of genes and genomes. Nucleic Acids Res 28: 27-30.

9. Saxena R, Voight BF, Lyssenko V, Burtt NP, de Bakker PI, et al. (2007) Genome-wide association analysis identifies loci for type 2 diabetes and triglyceride levels. Science 316: 1331-1336.

10. Scott LJ, Mohlke KL, Bonnycastle LL, Willer CJ, Li Y, et al. (2007) A genome-wide association study of type 2 diabetes in Finns detects multiple susceptibility variants. Science 316: 1341-1345.

11. Wellcome Trust Case Control Consortium (2007) Genome-wide association study of 14,000 cases of seven common diseases and 3,000 shared controls. Nature 447: 661-678.

12. Zeggini E, Weedon MN, Lindgren CM, Frayling TM, Elliott KS, et al. (2007) Replication of genome-wide association signals in UK samples reveals risk loci for type 2 diabetes. Science 316: 1336-1341.

13. Price AL, Patterson NJ, Plenge RM, Weinblatt ME, Shadick NA, et al. (2006) Principal components analysis corrects for stratification in genome-wide association studies. Nat Genet 38: 904-909.
