# Supplementary material for: A Phenomics-Based Strategy Identifies Loci on APOC1, BRAP, and PLCG1 Associated with Metabolic Syndrome Phenotype Domains
Source: PLoS Genet. 2011 Oct 13;7(10):e1002322. doi: 10.1371/journal.pgen.1002322 (PMC3192835; doi:10.1371/journal.pgen.1002322)
Supplement: Text S2 — Participating studies. (DOC) [file pgen.1002322.s024.doc]

Supplemental Methods

Studies of unrelated individuals

Suppose that we have a total of n subjects, K phenotypes (principal components), and p covariates. For $i = 1, \ldots, n$ and $k = 1, \ldots, K$, let Y_{ki} be the kth phenotype of the ith subject. For $i = 1, \ldots, n$ and $j = 1, \ldots, p$, let X_{ji} be the jth covariate of the ith subject. Then the data on phenotypes and covariates can be represented as $(Y_{1i}, \ldots, Y_{Ki}, X_{1i}, \ldots, X_{pi})$ $(i = 1, \ldots, n)$, where $X_{1i} = 1$.

For $i = 1, \ldots, n$, let G_i denote the number of minor alleles (or the imputed dosage) at a particular test locus. We assume the following linear regression models:

$$Y_{ki} = \beta_k^{\mathrm{T}} X_i + \gamma_k G_i + \epsilon_{ki} \quad (k = 1, \ldots, K; i = 1, \ldots, n),$$

where $X_i = (X_{1i}, \ldots, X_{pi})^{\mathrm{T}}$, β_k is the set of regression parameters associated with the covariates, γ_k is the regression parameter associated with the SNP genotype, and the ϵ_{ki} are random errors, which are potentially correlated among the K phenotypes.

To accommodate missing data, we let ξ_{ki} indicate, by the values 1 versus 0, whether Y_{ki} is observed or missing, and let ψ_i indicate, by the values 1 versus 0, whether G_i is observed or missing. It is assumed that covariates are completely observed.

Under the null hypothesis that $\gamma_k = 0$, the score function for γ_k is

$$U_k = \sum_{i=1}^{n} \xi_{ki} \psi_i (Y_{ki} - \widehat{\beta}_k^{\mathrm{T}} X_i) G_i,$$

where

$$\widehat{\beta}_k = \left(\sum_{i=1}^{n} \xi_{ki} X_i X_i^{\mathrm{T}} \right)^{-1} \sum_{i=1}^{n} \xi_{ki} Y_{ki} X_i.$$

Define the score vector

$$U = \begin{bmatrix} U_1 \\ \vdots \\ U_K \end{bmatrix}.$$

The covariance matrix of U can be estimated by $V = (A_{GG} - A_{GX}^{\mathrm{T}} A_{XX}^{-1} A_{GX}) C$, where

$$A_{GG} = \sum_{i=1}^{n} \psi_i G_i^2,$$

$$A_{GX} = \sum_{i=1}^{n} \psi_i G_i X_i,$$

$$A_{XX} = \sum_{i=1}^{n} X_i X_i^{\mathrm{T}},$$

$$C = \begin{bmatrix} C_{11} & \dots & C_{1K} \\ \vdots & \vdots & \vdots \\ C_{K1} & \dots & C_{KK} \end{bmatrix},$$

and

$$C_{kl} = n^{-1} \sum_{i=1}^{n} \xi_{ki} \xi_{li} (Y_{ki} - \widehat{\beta}_k^{\mathrm{T}} X_i)(Y_{li} - \widehat{\beta}_l^{\mathrm{T}} X_i), \quad k, l = 1, \dots, K.$$

To test the global null hypothesis that $\gamma_1 = \gamma_2 = \ldots = \gamma_K = 0$, we form the test statistic

$$Q = U^{\mathrm{T}} V^{-1} U,$$

which is referred to the chi-squared distribution with K degrees of freedom. Since $V = (A_{GG} - A_{GX}^{\mathrm{T}} A_{XX}^{-1} A_{GX})C$, we can also express the test statistic as

$$Q = \frac{U^{\mathrm{T}} C^{-1} U}{A_{GG} - A_{GX}^{\mathrm{T}} A_{XX}^{-1} A_{GX}}.$$

Note that $\widehat{\beta}_k$ $(k = 1, \dots, K)$, A_{XX} and C do not depend on the SNP genotypes and thus need to be calculated only once (before looping through all the SNPs). For each SNP, we calculate U, A_{GG} and A_{GX}, which are combined with the previously calculated A_{XX}^{-1} and C^{-1} to yield Q.

Family studies

Suppose that we have a total of n families, with n_i members in the ith family. The data on phenotypes and covariates can be represented as $(Y_{1ij}, \dots, Y_{Kij}, X_{1ij}, \dots, X_{pij})$ $(i = 1, \dots, n; j = 1, \dots, n_i)$, where Y_{kij} is the kth phenotype for the jth member of the ith family, X_{lij} is the lth covariate for the jth member of the ith family, with $X_{1ij} = 1$.

For $i = 1, \dots, n$ and $j = 1, \dots, n_i$, let G_{ij} denote the number of minor alleles (or the imputed dosage) which the jth member of the ith family carries at a particular test locus. We assume the following (marginal) linear regression models:

$$Y_{kij} = \beta_k^{\mathrm{T}} X_{ij} + \gamma_k G_{ij} + \epsilon_{kij} \quad (k = 1, \dots, K; i = 1, \dots, n; j = 1, \dots, n_i),$$

where $X_{ij} = (X_{1ij}, \dots, X_{pij})^{\mathrm{T}}$, β_k is the set of regression parameters associated with the covariates, γ_k is the regression parameter associated with the SNP genotype, and the ϵ_{kij} are random errors, which are potentially correlated among the K phenotypes and among the family members.

To accommodate missing data, we let ξ_{kij} indicate, by the values 1 versus 0, whether Y_{kij} is observed or missing, and let ψ_{ij} indicate, by the values 1 versus 0, whether G_{ij} is observed or missing. It is assumed that covariates are completely observed.

We adopt the GEE approach with the independence working assumption (Liang and Zeger, 1986) such that the family structures are arbitrary and the correlations among family members are estimated emprically. Under the null hypothesis that $\gamma_k = 0$, the score function for γ_k (under the working independence assumption) is

$$U_k = \sum_{i=1}^{n} \sum_{j=1}^{n_i} \xi_{kij} \psi_{ij} (Y_{kij} - \widehat{\beta}_k^{\mathrm{T}} X_{ij}) G_{ij},$$

where

$$\widehat{\beta}_k = \left(\sum_{i=1}^{n}\sum_{j=1}^{n_i} \xi_{kij} X_{ij} X_{ij}^{\mathrm{T}}\right)^{-1} \sum_{i=1}^{n}\sum_{j=1}^{n_i} \xi_{kij} Y_{kij} X_{ij}.$$

Define the score vector

$$U = \begin{bmatrix} U_1 \\ \vdots \\ U_K \end{bmatrix}.$$

By the arguments of Liang and Zeger (1986), the covariance matrix of U can be estimated by

$$V = \begin{bmatrix} V_{11} & \dots & V_{1K} \\ \vdots & \vdots & \vdots \\ V_{K1} & \dots & V_{KK} \end{bmatrix},$$

where

$$V_{kl} = \sum_{i=1}^{n} U_{ki} U_{li} \quad (k, l = 1, \dots, K),$$

$$U_{ki} = \sum_{j=1}^{n_i} \xi_{kij} (Y_{kij} - \widehat{\beta}_k^{\mathrm{T}} X_{ij})(\psi_{ij} G_{ij} - A_{GX}^{\mathrm{T}} A_{XX}^{-1} X_{ij}) \quad (k = 1, \dots, K; i = 1, \dots, n),$$

$$A_{GX} = \sum_{i=1}^{n}\sum_{j=1}^{n_i} \psi_{ij} G_{ij} X_{ij},$$

$$A_{XX} = \sum_{i=1}^{n}\sum_{j=1}^{n_i} X_{ij} X_{ij}^{\mathrm{T}}.$$

To test the global null hypothesis that $\gamma_1 = \gamma_2 = \ldots = \gamma_K = 0$, we form the test statistic

$$Q = U^{\mathrm{T}} V^{-1} U,$$

which is referred to the chi-squared distribution with K degrees of freedom. Note that $\widehat{\beta}_k$ $(k = 1, \dots, K)$ and A_{XX} do not depend on the SNP genotypes and thus need to be calculated only once (before looping through all the SNPs).

Meta-analysis

We wish to combine results from L independent studies. For $l = 1, \dots, L$, let U_l and V_l denote the score vector U and the covariance matrix V from the lth study. We form the test statistic

$$Q = \left\{\sum_{l=1}^{L} U_l\right\}^{\mathrm{T}} \left\{\sum_{l=1}^{L} V_l\right\}^{-1} \left\{\sum_{l=1}^{L} U_l\right\},$$

which is again referred to the chi-squared distribution with K degrees of freedom. Note that $\sum_{l=1}^{L} U_l$ is the score statistic in the joint analysis of the individual-level data of the L studies and is asymptotically equivalent to the familiar inverse-variance estimator. For

meta-analysis, K pertains to the total number of distinct phenotypes (principal components), some of which may be missing in some studies. (For $L = 2$, we may have 4 phenotypes that are common between the two studies, 2 phenotypes that are measured in the first study and 3 phenotypes that are measured in the second study. Then $K = 9$.)

References

Liang, K.-Y., and Zeger, S. L. (1986). Longitudinal data analysis using generalized linear models. *Biometrika*, **73**, 13-22.
